# Quantification of Fosfomycin in Combination with Nine Antibiotics in Human Plasma and Cation-Adjusted Mueller-Hinton II Broth via LCMS

**DOI:** 10.3390/antibiotics11010054

**Published:** 2022-01-02

**Authors:** Kelvin Kau-Kiat Goh, Wilson Ghim-Hon Toh, Daryl Kim-Hor Hee, Edwin Zhi-Wei Ting, Nathalie Grace Sy Chua, Farah Iffah Binte Zulkifli, Li-Jiao Sin, Thuan-Tong Tan, Andrea Lay-Hoon Kwa, Tze-Peng Lim

**Affiliations:** 1 Department of Pharmacy, Singapore General Hospital, Outram Road, Singapore 169608, Singapore; kelvin.goh.kau.kiat@sgh.com.sg (K.K.-K.G.); wilsontgh113@hotmail.com (W.G.-H.T.); nathalie.grace.sy.chua@sgh.com.sg (N.G.S.C.); farah.iffah.zulkifli@sgh.com.sg (F.I.B.Z.); sin.li.jiao@sgh.com.sg (L.-J.S.); 2 SingHealth Duke-NUS Pathology Academic Clinical Programme, 8 College Road, Singapore 169857, Singapore; 3 Shimadzu (Asia Pacific) Pte Ltd., 79 Science Park Dr, #02-01/08 Cintech IV, Singapore 118264, Singapore; zhiwei@shimadzu.com.sg (E.Z.-W.T.); daryl_hee@cgh.com.sg (D.K.-H.H.); 4 SingHealth Duke-NUS Medicine Academic Clinical Programme, 8 College Road, Singapore 169857, Singapore; tan.thuan.tong@singhealth.com.sg; 5 Department of Infectious Diseases, Singapore General Hospital, Outram Road, Singapore 169608, Singapore; 6 Emerging Infectious Diseases Program, Duke-NUS Medical School, 8 College Road, Singapore 169857, Singapore

**Keywords:** fosfomycin, LCMS/MS, antibiotics, plasma, TDM, validation

## Abstract

Fosfomycin-based combination therapy has emerged as an attractive option in our armamentarium due to its synergistic activity against carbapenem-resistant Gram-negative bacteria (CRGNB). The ability to simultaneously measure fosfomycin and other antibiotic drug levels will support in vitro and clinical investigations to develop rational antibiotic combination dosing regimens against CRGNB infections. We developed an analytical assay to measure fosfomycin with nine important antibiotics in human plasma and cation-adjusted Mueller–Hinton II broth (CAMHB). We employed a liquid-chromatography tandem mass spectrometry method and validated the method based on accuracy, precision, matrix effect, limit-of-detection, limit-of-quantification, specificity, carryover, and short-term and long-term stability on U.S. Food & Drug Administration (FDA) guidelines. Assay feasibility was assessed in a pilot clinical study in four patients on antibiotic combination therapy. Simultaneous quantification of fosfomycin, levofloxacin, meropenem, doripenem, aztreonam, piperacillin/tazobactam, ceftolozane/tazobactam, ceftazidime/avibactam, cefepime, and tigecycline in plasma and CAMHB were achieved within 4.5 min. Precision, accuracy, specificity, and carryover were within FDA guidelines. Fosfomycin combined with any of the nine antibiotics were stable in plasma and CAMHB up to 4 weeks at −80 °C. The assay identified and quantified the respective antibiotics administered in the four subjects. Our assay can be a valuable tool for in vitro and clinical applications.

## 1. Introduction

Fosfomycin is an old broad-spectrum antibiotic with bactericidal activity against Gram-positive and Gram-negative bacteria but has recently gained renewed interest in treating multidrug-resistant (MDR) bacterial infections [1,2]. In addition, intravenous fosfomycin has been increasingly used in combination with other antibiotics as last-line therapy for carbapenem-resistant Gram-negative bacteria (CRGNB) infections due to increased rates of resistance emergence, acquired resistance, and heteroresistance with fosfomycin monotherapy [3,4,5]. Overall, antibiotic combination therapy is rapidly becoming a viable alternative to alleviate the burden of lack of effective antibiotics remaining in our antibiotic armamentarium. In addition, combination drug therapy allows repositioning or repurposing of approved drugs to give clinicians alternate therapeutic solutions for multidrug-resistant infections that would otherwise have been rendered untreatable if subjected to monotherapy [6]. Notably, this also meant that drug assays that can simultaneously measure multiple antibiotic classes in a single run are gaining traction as combination-based drug therapies grow increasingly popular. Such multiplexed assays are necessary to optimize fosfomycin-based combination dosing regimens for different CRGNB infections in various clinical scenarios [7]. Therefore, there is an emerging need for a fast, sensitive, and robust analytical method for quantifying fosfomycin and other antibiotics in human biological samples and in vitro testing medium (i.e., cation-adjusted Mueller Hinton II media) to facilitate clinical translation.

Multiplexed assays are also highly enabling when applied to therapeutic drug monitoring to report multiple drug levels with minimal turn-around time, especially when patients may be on polypharmacy resulting from various illnesses. Furthermore, optimizing dose to treat critically ill patients is challenging due to their severely altered and variable antibiotic pharmacokinetics caused by altered fluid status, varying serum albumin concentrations and renal and hepatic function, and microvascular failure [8]. Conventional strategies for dosing with antibiotics in critically ill patients that rely on general product information are less-prone to consistently achieve the pharmacokinetic/pharmacodynamic (PK/PD) targets associated with maximum antibiotic activity, which in turn elevates the risk of clinical failure or development of resistance, or both [8]. Therefore, antibiotic dosing in the intensive care unit should take an individualized approach whereby clinicians consider the antibiotic’s minimum inhibitory concentration for the infecting pathogen and derive the dosing regimen that is likely to obtain the PK/PD target to ensure the highest likelihood of success of positive clinical outcomes. Hence, an accurate and fast multiplex drug assay is paramount in the continual efforts to finetune drug dosing, especially in individualized treatments. 

Over the past five years, mass spectrometry-based methods to measure fosfomycin in various matrices such as plasma [9,10,11], plasma dried spots [12], growth media [13], and urine [11,14,15] have been reported to support PK/PD optimization of fosfomycin therapy. Interestingly, these methods solely measure fosfomycin. There are no available multiplex antibiotic assays that measure fosfomycin in combination with other antibiotics, despite recently reported multiplex assays for other various classes of antibiotics [16,17,18]. 

Our study aims to develop a liquid chromatography-tandem mass spectrometry (LCMS/MS) method to measure fosfomycin and nine important antibiotics concurrently in human plasma and cation-adjusted Mueller-Hinton II broth (CAMHB). The list of antibiotics to be measured with fosfomycin will cover a wide antibiotic class range, including beta-lactams, fluoroquinolones, and glycylcyclines used to treat current MDR or CRGNB infections in our local population in Singapore. For the assay optimization, an emphasis on a short runtime (<5 min) to potentially enable high throughput sampling under routine clinical laboratory conditions, a unified sample preparation protocol for multiple matrices (plasma and CAMHB), and an extensive concentration testing range for all antibiotics. Antibiotics included in our method (aztreonam, cefepime, ceftazidime-avibactam, ceftolozane-tazobactam, doripenem, levofloxacin, meropenem, piperacillin-tazobactam, and tigecycline) are commonly prescribed to infected critically ill patients who are at risk with or have multidrug-resistant infections [19] or evaluated in PK/PD studies in combination with fosfomycin. After the method was established, we validated the assay according to FDA guidelines [20]. In addition, we successfully applied the assay in a prospective clinical study [21] to evaluate the clinical feasibility and utility of β-lactam TDM in critically-ill patients and report our findings in four subjects on fosfomycin-based combination drug therapy. 

## 2. Results

### 2.1. Assay Validation

#### 2.1.1. Specificity, Selectivity, and Carryover

No interfering peaks or contamination of isotope variants of any compound were observed at the indicated retention times for the ten antibiotics (twelve compounds) in CAMHB and the six different non-spiked human plasma samples (Supplementary Materials Figures S1–S12). The multiple reaction monitoring (MRM) transitions (Supplementary Materials Table S1) were selective and specific for all the compounds and their internal standards in plasma and CAMHB. Most compounds exhibit no or minimal carryovers [<5% of the lower limit-of-quantification (LLOQ)] except for piperacillin, levofloxacin, and avibactam (>10% of LLOQ). All internal standards of any compound had no or minimal carryover (<0.15%) as well. Therefore, the assay passed acceptance criteria since there was no significant carryover present which was defined by having peak areas of the analyte and internal standard in the blank sample to be less than 20% of the area of the LLOQ or 5% of the area of the internal standard, respectively (Table S2).

#### 2.1.2. Calibration Curve, Accuracy, Precision LLOQ, and Limit of Detection (LOD)

A total of 8 calibrator levels and 3 (quality control) QC levels were validated across the concentration range shown in Table 1. All compounds spiked in plasma and CAMHB exhibited good linearity (r^2^ > 0.999) across the tested concentration range based on linear regression using 1/x weighting. As FDA does not provide any recommendations on the selection of nominal concentrations for the QC levels, we have adapted our nominal concentration for the low QC (LQC), medium QC (MQC), and high QC (HQC) based on guidelines from the European Medicine Agency (EMA) [22]. LQC was set at 8.3x of LLOQ. This selection accounts for situations in which the LLOQ may potentially fail. Considering such a scenario, calibrator 2 will then be made the next LLOQ and the calibration should still maintain 3 QCs. Hence, nominal concentration for LQC was set around the mid-point between calibrators 2 and 3. MQC and HQC were set at 37.5% and 87.5% of the full calibration range, respectively. LOD for all compounds in plasma and CAMHB are summarized in Table S3. In addition, all stock solutions for the compounds were found to be stable when kept at −80°C for 32 days (Table S4). Results for within-run and between-run accuracy and precision for the compounds spiked in plasma and CAMHB were summarized in Table 2 and Table 3, respectively. All within-run, between-run mean accuracies and precision for all compounds in plasma and CAMHB except tigecycline in plasma (84.0%) passed acceptance criteria. The LLOQ was validated at calibrator level 1, while the upper-limit-of quantification (ULOQ) was validated at calibrator level 8. All calibration curves require a minimum of six non-zero calibrators to pass acceptance criteria [20].

#### 2.1.3. Matrix Factor (MF) and Internal Standard-Normalized Matrix Factor (IS-nMF) 

Average MF across all compounds spiked in plasma ranged from 22.0%–224.5% (Table S5). Ion suppression was observed for meropenem, doripenem, avibactam, cefepime, and fosfomycin, while ion enhancement was observed in tigecycline and levofloxacin. Average MF across all compounds spiked in CAMHB ranged from 17.6%–226.2% (Table S6). Ion suppression in CAMHB was observed for meropenem, doripenem, levofloxacin, avibactam, aztreonam, ceftolozane, tazobactam, and fosfomycin. Ion enhancement was observed for tigecycline. The stable-isotope labeled IS effectively compensated for the matrix effect demonstrated by IS-nMF, ranging between 89.3%–114.7% (Tables S5 and S6).

### 2.2. Stability Studies of Fosfomycin with Other Antibiotics

Antibiotic-spiked plasma/CAMHB samples kept under autosampler conditions for 7 h were stable (86.5%–113.7%, Tables S7 and S8) except for piperacillin MQC, which narrowly missed the cut-off (84.0%). However, all plasma and CAMHB samples were deemed stable for up to 15 h (86.8%–114.1%, Tables S9 and S10).

In combination, fosfomycin spiked with the respective antibiotics were stable in plasma at −30 °C and −80 °C for up to 4 weeks (86.1%–112.6%, Tables S11–S14). However, for CAMHB samples stored at −30 °C, instability was observed in doripenem (2 weeks) and cefepime, piperacillin, aztreonam, meropenem, doripenem, and avibactam (4 weeks) (60.1%–110.1%, Tables S15 and S16). In contrast, CAMHB samples stored up to 4 weeks were stable for all tested antibiotics at −80°C (87.5–111.6%, Tables S17 and S18).

### 2.3. Application to a Pilot Clinical Feasibility Study and a Case-Series

**Figure 1:**
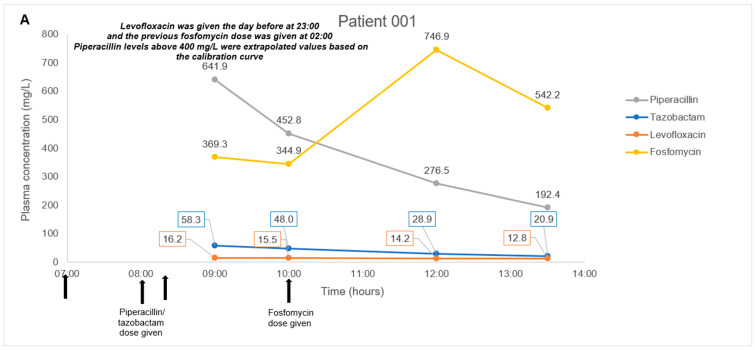

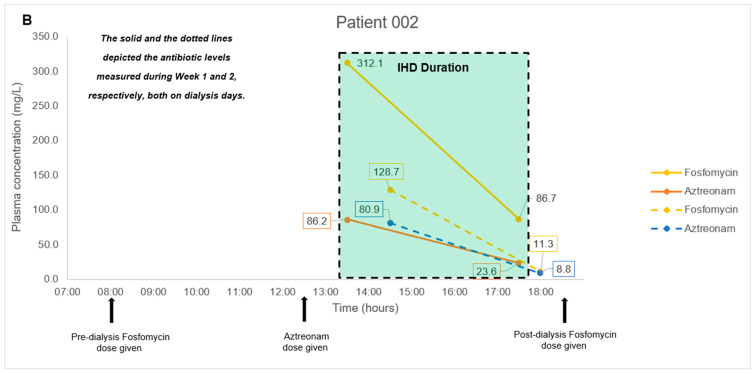

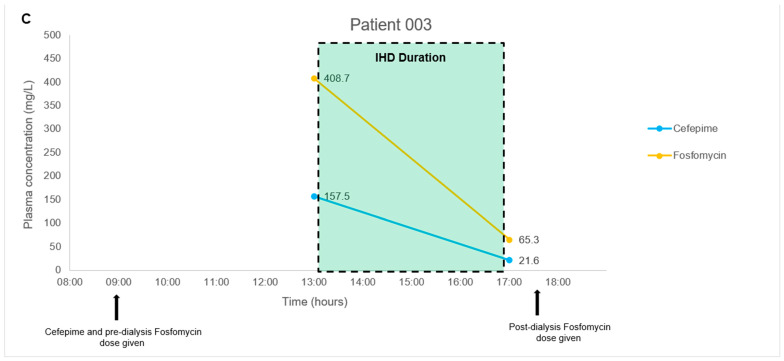

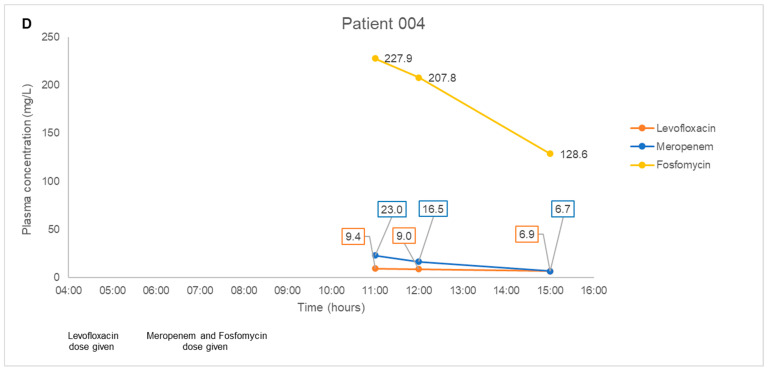
(**A**–**D**). Plasma concentration-time courses of fosfomycin and the respective combination antibiotics in the four patients. Black arrows depict antibiotic administration. Dialysis treatment duration is depicted by box size. IHD, intermittent hemodialysis.

The antibiotic combination regimens and basic demographics for each patient are presented in Table 4. Mean total plasma concentrations of fosfomycin and respective antibiotics measured in each patient are presented in Figure 1. Plasma samples obtained from four patients were measured in triplicates and the mean concentration was derived ranging from 11.3–746.9 mg/L for fosfomycin. Mean levofloxacin concentrations ranged from 6.9–16.2 mg/L in Patient 001 and 004. Mean piperacillin and tazobactam concentrations ranged from 192.4–641.9 mg/L and 20.9–58.3 mg/L, respectively, in Patient 001. Mean aztreonam, cefepime, and meropenem concentrations ranged from 8.8–86.2 mg/L, 21.6–157.5 mg/L, and 6.7–23.0 mg/L in Patient 002, 003, and 004, respectively. For all patient samples measuring fosfomycin, aztreonam, cefepime, levofloxacin, piperacillin, and tazobactam, the results were within 3.9%, 4.8%, 8.1%, 2.1%, 4.6, and 6.2% precision, respectively. 

Plasma quality control samples tested within batches of these study samples were prepared at the following concentrations of fosfomycin: (25, 750 and 80 mg/L), aztreonam: (5, 150 and 350 mg/L), cefepime: (2.5, 75 and 175 mg/L), levofloxacin: (0.5, 15 and 35 mg/L), piperacillin: (5, 150 and 175 mg/L) and tazobactam: (1.25, 37.5 and 87.5 mg/L). For all plasma quality control samples, fosfomycin was within 1.9% precision and 98–107% accuracy, aztreonam was within 6.2% precision and 99–105% accuracy, cefepime was within 7.6% precision and 100–103% accuracy, levofloxacin was within 2.5% precision and 99–103% accuracy, piperacillin was within 1.8% precision, and 92–103% accuracy and tazobactam was within 5.6% precision and 98–111% accuracy (See Table 5). 

## 3. Discussion

Hydrophilic interaction liquid chromatography (HILIC) is typically employed among the reported fosfomycin-based LCMS/MS methods due to good compatibility for compounds such as fosfomycin which is hydrophilic and polar [9,11,13,14]. Hence, it allows excellent retention of the compound, resulting in sharp peaks and good separation. In contrast, other common antibiotics such as β-lactams [23,24] and fluoroquinolones [25] were developed utilizing mostly reverse-phase liquid chromatography (RPLC) C18 columns that were designed for hydrophobic and non-polar compounds, thus, enabled good separation and sufficient retention. However, because of the different chemistries of the chromatography columns used, it is challenging to retain both fosfomycin (a very polar molecule) and the other less polar antibiotics (relative to fosfomycin) using either HILIC or RPLC (C18) columns. While we optimized our chromatography for compound retention during various column trials, we found that using either a 2.1 × 100 mm T3 C18 or C8 column retained fosfomycin sufficiently to be captured on the chromatogram together with the other antibiotics. Our observation was in agreement with the findings of Fuad J. Naser et al. where they described the deficiency of the conventional approach of using HILIC and RPLC (C18) for use in untargeted metabolomics due to the poor ability of both existing chromatographic methods to separate and detect many metabolites in the range of intermediate polarity, which they have termed as “semipolar” [26]. They evaluated the use of both T3 C18 and C8 columns and found that it could extend compound coverage in addition to existing HILIC and RPLC (C18)-based methods by detecting such medium or intermediate polar compounds. In our case, we describe the other antibiotics barring fosfomycin as “semi-polar” and were not well-retained on the HILIC column. 

**Figure 2:**
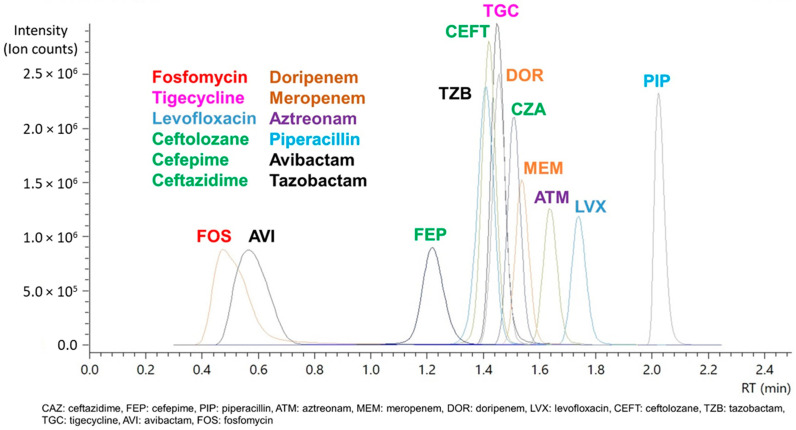
Chromatogram of each antibiotic compound and β-lactamase inhibitor (depicted in black) using a 2.1 × 50 mm C8 column.

Further optimization between both the T3 C18 and C8 column led to the use of the latter after trials on gradient elution further improved separation and retention for fosfomycin and the nine study antibiotics, and the use of a shorter 2.1 × 50 mm column enabled the method to achieve a short runtime of 4.5 min including column equilibration for all the compounds (Figure 2). While our optimized method was able to elute the last compound, piperacillin, by 2.15 min, an additional time (up to 2 min) was included for flushing and re-equilibration to ensure minimal carryover and robust performance. This is important as it contributes to conserving our column despite the lack of sample cleanup, which is most optimally done using solid-phase extraction (SPE) cartridges to clean and concentrate sample extracts prior to injection into the MS. Sample preparation was centered around the most straightforward and cheapest option available to us: acetonitrile to precipitate proteins from the plasma or CAMHB samples. Despite using simple protein precipitation, the column has achieved over 7000 injections on plasma samples using this method without replacement or any retention drift and blockage issues.

In addition, the gradient elution was accomplished using formic acid in water or acetonitrile as mobile phases in the absence of any salt buffers. We find this feature a significant factor in enhancing the robustness and ruggedness of the method as previous experience has shown that salt buffers increase the chances of salt precipitation in our mobile phase lines, resulting in clogging incidents. Clogged lines or columns can result in reduced consistency in chromatography, more frequent and expensive column replacements, and significant operation time loss due to increased maintenance and repairs. These can be mitigated by consistent flushing with non-buffered water after runs and monitoring of back pressure. Moreover, eliminating salt buffers from our mobile phase has brought much simplicity to our daily maintenance when operating the method. Overall, the method developed with the sample preparation allows us to minimize the costs of running and maintaining the equipment when running at high capacity.

Selectivity tests have shown that our LCMS/MS method can differentiate the analytes and the IS from endogenous and exogenous compounds in plasma and CAMHB matrices based on their specific multiple reaction monitoring (MRM) mass transitions. In complex matrices such as plasma, matrix effects due to endogenous compounds can enhance or suppress the signal intensity of the target compounds, leading to inaccurate representations of the actual drug concentration. This was also observed in our study, where we found ion suppression of meropenem, doripenem, avibactam, cefepime, and fosfomycin (strongest) spiked in plasma (Table S5). In CAMHB, the same antibiotics were suppressed with the addition of levofloxacin, aztreonam, ceftolozane, and tazobactam (Table S6). This would suggest that these antibiotics in CAMHB would be more challenging to measure accurately without considering matrix effects, and more caution should be exercised when developing an assay for them. Our strategy to use stable-isotope variants of the antibiotics as internal standards were able to circumvent and negate the varying influence by the matrix, resulting in the good linear response that gives rise to our good accuracies and precision in our calibration curve, which are within the acceptable limits as determined by FDA guidance [20]. 

In our pilot study on four patients, we did not encounter any complex issues on assay interference. Furthermore, the multiple antibiotics identified in each patient’s plasma samples correlated to the antibiotic combination dosing regimens observed clinically. Thus, even though antibiotic pharmacokinetics is not the main focus of the pilot study, the fluctuating antibiotic concentrations observed in our case series of four patients were consistent with the expected individual pharmacokinetic profiles. In Patients 001 and 004 with normal renal function, we observed a consistent decrease in fosfomycin, piperacillin-tazobactam, meropenem, and levofloxacin post-administration; and detected a steady increase in fosfomycin concentrations upon redosing in Patient 001. In Patient 002 and 003 with impaired renal function, we observed a substantial decrease in all antibiotic concentrations measured post intermittent hemodialysis. These observations suggested that our method is likely to be highly robust and have minimal assay interference from endogenous compounds. This key feature will be essential when measuring antibiotic levels in critically-ill patients who are often on multiple drugs.

Based on the FDA recommended criteria [20] for stability as defined by compound loss not exceeding 15%, our stability study has shown acceptable values for any of the tested antibiotic combinations with fosfomycin for up to 15 h at 4 °C, apart from piperacillin (MQC-piperacillin failed FDA criteria at the 7-h time point). Our findings concurred with Forier et al., who reported limited piperacillin stability in sputum on ice [27]. Long-term storage of plasma samples was demonstrated for up to 4 weeks either at −30 °C or −80 °C; CAMHB samples can be stored at −80 °C for up to 4 weeks. Thus, for all antibiotic combinations with fosfomycin in plasma and CAMHB, the recommended storage condition is at −80 °C for up to 4 weeks prior to analysis. If −80 °C storage access is not available, plasma samples can be kept stable at −30 °C prior to analysis for up to 4 weeks. However, we noted that the piperacillin stability data at 2-week and 4-week intervals (~86–88%) in plasma were close to the FDA cut-off criteria (±15%). Therefore, for added assurance, we suggest analyzing piperacillin-containing samples in plasma as soon as logistics permit or no more than 2 weeks of storage after taking samples. 

This study has some limitations. Firstly, we did not validate the multiple freeze-thaw cycle stability of such fosfomycin-based combination samples in either matrix. As a small sample volume of 20 uL was required in our assay method, the practice of preparing multiple aliquots from each plasma sample (~1 mL) was feasible to accommodate for reruns. As a result, it became routine for us to have multiple replicate aliquots, and all analyzed samples were never subjected to more than the initial freeze-thaw. Nonetheless, freeze-thaw stability data may benefit those who do not have the practice of keeping multiple aliquots or may be further restricted by sample storage. Next, an unexpected limitation was observed in our concentration range despite expanding the upper limit by almost twice as compared to recent methods. To quote a few specific examples, our fosfomycin upper limit was doubled to 2000 mg/L as compared with 1000 mg/L from Martens-Lobenhoffer et al. and Wijma et al. [9,11] and piperacillin upper limit was also doubled to 400 mg/L as compared with 160 mg/L from Decosterd et al. [18] and 200 mg/L from a method by Barco et al. [17]. Unexpectedly, the study sample from Patient 1 exhibited piperacillin levels of ~641.9 mg/L and ~452.8 mg/L that exceeded our upper limit (Figure 1A). These values were extrapolated from our calibration curve and may minimize the confidence of the reported value as it does not fall within the validated range. We recommend that future methods be expanded even further as reports of clinically achievable drug levels in plasma can increase dramatically. Lastly, while the method is validated for drug spiked sterile CAMHB samples, this study has not demonstrated any in vitro applications using actual run samples. Factors such as the metabolites from bacteria presence and prolonged contact with plastic paraphernalia of the in vitro setup may introduce possible chromatographic or ionization interference that will affect the assay performance, and is yet to be studied. We hope to address this in future in vitro PK/PD studies on fosfomycin-based combinations against MDR bacteria isolates. 

Fosfomycin-based combination regimens have been proposed as a possible treatment strategy against CRGNB infections instead of monotherapy due to its propensity to develop resistance rapidly in vitro [28,29]. These useful treatment alternatives include ceftolozane/tazobactam [30], meropenem [31], and ceftazidime/avibactam respectively, with fosfomycin [32,33]. In our pilot study, our patients were prescribed a variety of fosfomycin-based combination therapy for their CRGNB infections due to the extensively-drug resistant phenotype of the infecting bacteria; and polymyxin B was not a suitable monotherapy treatment option clinically [34]. The fosfomycin combination stability data will be helpful when conducting PK/PD modeling studies evaluating fosfomycin-based antibiotic combinations. The unique combination therapy options were derived from a real-time in vitro antibiotic combination test that identifies bactericidal antibiotic combinations against the infecting bacteria within 48 h of starting the test [19]. Given the growing importance of combination therapy entailing two or more antibiotics, the long-term stability demonstrated for all antibiotic combinations with fosfomycin in plasma and CAMHB will be helpful when conducting PK/PD modeling and clinical studies evaluating fosfomycin-based antibiotic combinations.

## 4. Materials and Methods

### 4.1. Chemicals

Fosfomycin-disodium, ceftazidime-pentahydrate, aztreonam, meropenem-trihydrate, tazobactam-sodium, [^2^H_9_]-tigecycline, and fosfomycin-^13^C_3_-benzylamine were purchased from Toronto Research Chemicals (North York, ON, Canada). Cefepime-hydrochloride-monohydrate, piperacillin-sodium, levofloxacin, and tigecycline were purchased from Sigma-Aldrich (St. Louis, MO, USA). Doripenem was purchased from Shionogi & Co., Ltd. (Osaka, Japan). Ceftolozane-sulphate was purchased from MicroConstants (San Diego, CA, USA). Avibactam-sodium was provided by Pfizer Inc (New York, NY, USA). [^2^H_6_]-Ceftazidime-ditrifluoroacetate, [^13^C,^2^H_3_]-cefepime-sulphate, [^2^H_5_]-piperacillin-sodium, [^2^H_6_]-aztreonam-formate, [^2^H_6_]-meropenem, [^2^H_5_]-doripenem, [^13^C,^2^H_3_]-levofloxacin, [^15^N_2_,^2^H_4_]-ceftolozane-trifluoroacetate, [^13^C_5_]-avibactam-sodium, and [^13^C_2_,^15^N_3_]-tazobactam-sodium were purchased from Alsachim (Illkirch-Graffenstaden, France). Water from a Milli-Q Gradient water system (Millipore, MA, Bedford, TX, USA), LC-MS grade formic acid (VWR, Radnor, PA, USA), and acetonitrile (Thermo Fisher Scientific, Waltham, MA, USA) were used for mobile phase preparation. Blank human plasma was purchased from Zen-Bio (Durham, NC, USA). CAMHB was purchased from Becton-Dickinson (BBL, Sparks, NV, USA). Calibration standard and stable-isotope antibiotic variant working solutions were freshly prepared to give the target concentration levels for eight calibrator levels and three separate QCs as described in Table 1. 

### 4.2. Sample Preparation for LCMS/MS Analysis

For 20 µL of plasma/CAMHB sample, 20 µL of water and 20 µL of stable-isotope as internal standard (IS) were added and vortexed. 180 µL of acetonitrile was added for protein precipitation and subsequently removed by centrifugation (12,700 rpm for 10 min at 25 °C). Finally, 15 µL of the supernatant was diluted with 225 µL formic acid in water (0.1% *v*/*v*), and 10 µL was injected for LCMS/MS analysis. For the calibration curve and QCs, 20 µL of the corresponding antibiotic standard working solution was added instead of water. 

### 4.3. LCMS/MS Conditions and Analysis

Samples were analyzed using a Nexera X2 LCMS 8060 system (Shimadzu, Kyoto, Japan). Chromatographic separation was achieved (See Figure 2) using a Zorbax Eclipse Plus C8 column (2.1 × 50 mm, 1.8 µm) (Agilent Technologies, Santa Clara, CA, USA) with gradient elution of water and acetonitrile containing 0.1% (*v*/*v*) formic acid as mobile phases A and B respectively at a flow rate of 0.3 mL/min over a 4.5 min program (see Supplementary Materials for details). Electrospray ionization employing positive and negative ion mode with multiple reaction monitoring was used for analyte quantification (Table S1). Data were acquired and analyzed using Shimadzu LabSolutions (Version 5.97).

### 4.4. Specificity, Selectivity, and Carryover 

Specificity and selectivity were performed by comparing blank plasma of six different sources spiked with their corresponding compounds at the LLOQ level or isotope variants. Carryover was assessed by injecting blank samples after the ULOQ sample. 

### 4.5. Accuracy, Precision, Lower Limit-of-Quantification (LLOQ), and Limit-of-Detection (LOD)

Within-run, between-run accuracy and precision were measured and compared using eight calibrator levels with six replicates per calibrator level on four different days, respectively. The lowest calibrator level determined within ±20% of nominal concentration was arbitrarily set as the LLOQ. LOD was determined by having signal-to-noise ratios above 3.3. Acceptance criteria for calibration curve are met when the mean concentrations and coefficient of variation (CV) of a minimum of six non-zero are within ±15% of the nominal concentration.

### 4.6. Matrix Factor (MF) and INTERNAL Standard-Normalized MF (IS-nMF)

The MF of each analyte was calculated using the ratio of mean peak area (MPA) of post-spiked plasma/CAMHB samples and from the direct injection of the same concentration of analyte in water and evaluated at three different concentrations of low QC (LQC), medium QC (MQC) and high QC (HQC). IS-nMF was calculated using the ratio of MPA of post-spiked plasma/CAMHB samples without IS and with IS and compared to the ratio of MPA of direct injection of the same concentration of analyte in water without IS and with IS. 

### 4.7. Stability Studies of Fosfomycin with Other Antibiotics

The stability of fosfomycin in combination with other antibiotics in plasma/CAMHB was evaluated under various operational conditions. Spiked plasma/CAMHB samples were evaluated at 7 h and 15 h in the autosampler at 4 °C. Long-term stability was evaluated up to 4 weeks of antibiotic-spiked plasma/CAMHB samples at LQC, MQC, and HQC concentrations stored at −30 °C and −80 °C. Acceptance criteria for stability are met when the mean concentrations and coefficient of variation (CV) are within ±15% of the nominal concentration.

### 4.8. Application to a Pilot Clinical Feasibility Study and a Case-Series

The assay method described in this paper has been applied to a pilot clinical feasibility study to evaluate the proportion of patients with sub-optimal β-lactam levels and the need for dose adjustments in infected patients with unpredictable pharmacokinetics [21]. We described a case series of four patients where they received in vitro guided antibiotic combination therapy using a fosfomycin-based combination regimen to treat a carbapenem-resistant Gram-negative bacteria infection [19]. These four patients were recruited into the BLAST-2 study as the combination regimen contains a β-lactam antibiotic. In this case series, two were undergoing intermittent hemodialysis, and two patients had normal renal function. In the two patients on hemodialysis, they were on a dosing regimen of 8 g of fosfomycin on dialysis days (3 g at least 4 h before dialysis session and 5 g after completion of dialysis) [35]. The remaining two patients received 8 g of fosfomycin given every 8 h as a prolonged infusion. 

Blood samples (2–3 mL from an indwelling catheter) were collected four times over a dosing interval of the β-lactam dosing regimen and centrifuged (10 min at 3000 rpm) (Eppendorf 5425R). The resultant plasma was stored (within 15 min of collection) at −80 °C until assay.

## 5. Conclusions

To the best of our knowledge, no reported assay has been developed and validated to simultaneously measure fosfomycin and other antibiotics. Our validated multiplexed antibiotic assay is robust, fast, and simple to operate and can serve as a valuable tool for studying fosfomycin alone and/or in combination with the other antibiotics in PK/PD models and possibly routine TDM implementation. 

## Figures and Tables

**Table 1:** Nominal concentrations used in calibrators and QCs for all antibiotic compounds in plasma and CAMHB.

Compound	CAZ	FEP	PIP	ATM	MEM	DOR	LVX	CEFT	TZB	TGC	AVI	FOF
Concentration (mg/L)												
1 (LLOQ)	0.6	0.3	0.6	0.6	0.6	0.3	0.06	0.6	0.15	0.15	0.15	3
2	1	0.5	1	1	1	0.5	0.1	1	0.25	0.25	0.25	5
3	10	5	10	10	10	5	1	10	2.5	2.5	2.5	50
4	50	25	50	50	50	25	5	50	12.5	12.5	12.5	250
5	100	50	100	100	100	50	10	100	25	25	25	500
6	200	100	200	200	200	100	20	200	50	50	50	1000
7	300	150	300	300	300	150	30	300	75	75	75	1500
8 (ULOQ)	400	200	400	400	400	200	40	400	100	100	100	2000
LQC	5	2.5	5	5	5	2.5	0.5	5	1.25	1.25	1.25	25
MQC	150	75	150	150	150	75	15	150	37.5	37.5	37.5	750
HQC	350	175	350	350	350	175	35	350	87.5	87.5	87.5	1750

CAZ: ceftazidime, FEP: cefepime, PIP: piperacillin, ATM: aztreonam, MEM: meropenem, DOR: doripenem, LVX: levofloxacin, CEFT: ceftolozane, TZB: tazobactam, TGC: tigecycline, AVI: avibactam, FOF: fosfomycin, LLOQ: lower limit-of-quantification, ULOQ: upper limit-of-quantification, LQC: low-quality control, MQC: mid-quality control; HQC: high-quality control.

**Table 2:** Within- and between-run accuracies of 12 compounds in plasma for LLOQ, ULOQ, LQC, MQC and HQC.

Compound	CAZ	FEP	PIP	ATM	MEM	DOR	LVX	CEFT	TZB	TGC	AVI	FOF
Accuracy (%)												
PLASMA												
Within-run												
LLOQ	94.5 (6.1)	93.4 (4.8)	108.7 (2.3)	104.2 (3.7)	108.4 (10.1)	105.4 (5.4)	114.8 (2.2)	105.1 (12.4)	106.2 (4.9)	116.5 (4.2)	99.6 (2.0)	106.2 (3.9)
ULOQ	96.8 (2.9)	98.8 (2.6)	102.6 (0.5)	101.1 (4.3)	101.3 (4.1)	104.4 (2.5)	104.0 (1.0)	103.0 (2.9)	102.7 (1.5)	102.4 (2.9)	101.2 (0.5)	102.3 (0.8)
LQC	96.2 (3.8)	101.2 (2.7)	101.0 (3.2)	100.7 (5.0)	96.5 (4.4)	98.4 (2.8)	88.9 (0.5)	94.4 (6.4)	94.6 (4.4)	84.0 ^a^ (4.8)	96.8 (1.0)	95.2 (2.3)
MQC	101.1 (3.6)	103.0 (3.3)	97.9 (0.7)	100.6 (2.6)	103.5 (4.4)	97.5 (3.8)	97.3 (0.3)	98.1 (4.1)	100.9 (2.7)	96.0 (2.5)	99.7 (0.5)	98.7 (0.9)
HQC	99.0 (3.9)	97.7 (0.6)	103.8 (0.7)	101.5 (3.0)	108.7 (3.6)	100.9 (2.3)	102.6 (0.8)	98.0 (2.4)	104.2 (2.3)	108.0 (2.4)	100.5 (0.5)	102.3 (1.1)
r^2^ value	0.9988453	0.9995205	0.9991706	0.9998476	0.9995018	0.998757	0.9986312	0.9994118	0.9994067	0.9985256	0.9998819	0.9996225
gradient	y = 0.79169x	y = 11.0991x	y = 0.21593x	y = 0.398052x	y = 0.45178x	y = 1.29971x	y = 0.22326x	y = 2.03945x	y = 1.04784x	y = 1.93816x	y = 1.17966x	y = 0.82013x
y-intercept	−0.000575	0.021203	−0.001160	−0.001583	−0.002272	−0.005883	−0.001841	−0.000458	−0.004811	−0.037635	0.000110	−0.003660
Between-run												
LLOQ	94.1 (6.0)	89.2 (4.5)	113.9 (2.9)	105.0 (9.2)	100.8 (8.2)	102.0 (5.4)	115.4 (2.1)	98.3 (7.0)	106.7 (8.4)	111.4 (3.1)	100.4 (2.1)	109.6 (2.1)
ULOQ	94.4 (2.6)	97.6 (5.7)	103.2 (1.7)	98.9 (1.4)	98.3 (1.9)	98.8 (1.2)	102.3 (1.2)	100.6 (3.1)	101.8 (1.0)	94.7 (3.3)	101.2 (0.6)	101.9 (1.1)
LQC	97.6 (3.7)	101.1 (4.4)	92.7 (2.4)	98.0 (3.4)	98.9 (4.1)	99.5 (2.9)	87.2 (1.6)	93.8 (4.0)	100.8 (2.5)	85.3 (2.7)	99.7 (1.1)	95.2 (2.7)
MQC	104.8 (2.9)	103.7 (4.7)	96.3 (1.6)	101.0 (2.7)	102.8 (6.3)	101.1 (2.8)	99.6 (0.6)	97.4 (3.2)	102.0 (1.4)	102.2 (3.0)	98.8 (0.8)	98.8 (1.7)
HQC	98.2 (3.4)	98.5 (5.3)	104.4 (1.7)	103.4 (1.9)	99.9 (2.7)	101.6 (3.4)	106.8 (0.9)	98.9 (3.2)	104.8 (1.0)	99.8 (2.9)	101.5 (0.6)	102.7 (1.5)
r^2^ value	0.9993882	0.9987315	0.9987859	0.9993404	0.9994486	0.9990514	0.9987863	0.9992368	0.9994406	0.9985435	0.9997051	0.9989627
gradient	y = 0.58647x	y = 9.58180x	y = 0.17279x	y = 0.38990x	y= 0.46060x	y = 1.17540x	y = 0.22065x	y = 1.64273x	y = 0.88387x	y = 1.51796x	y = 1.18068x	y = 1.14038x
y-intercept	0.001606	0.029099	−0.000765	−0.001317	−0.001073	−0.002664	−0.001884	−0.004770	−0.001693	−0.035293	−0.000473	−0.008392

Accuracy (%) is denoted by mean concentration over nominal concentration and is accepted if ±15% or ±20% (for LLOQ). LLOQ: Lower limit-of-quantification, ULOQ: Upper limit-of-quantification, LQC: Low QC, MQC:. Mid QC, HQC: High QC. CAZ: ceftazidime, FEP: cefepime, PIP: piperacillin, ATM: aztreonam, MEM: meropenem, DOR: doripenem, LVX: levofloxacin, CEFT: ceftolozane, TZB: tazobactam, TGC: tigecycline, AVI: avibactam, FOS: fosfomycin. Precision (%CV) is denoted in parenthesis. ^a^ Value not compliant with FDA guidelines.

**Table 3:** Within- and between-run accuracies of 12 compounds in CAMHB for LLOQ, ULOQ, LQC, MQC and HQC.

Compound	CAZ	FEP	PIP	ATM	MEM	DOR	LVX	CEFT	TZB	TGC	AVI	FOF
Accuracy (%)												
CAMHB												
Within-run												
LLOQ	96.2 (5.6)	87.3 (4.0)	113.7 (0.8)	113.8 (5.0)	113.9 (1.0)	98.9 (7.1)	112.3 (2.4)	106.9 (8.4)	103.0 (9.9)	115.3 (2.1)	103.1 (4.2)	109.9 (2.2)
ULOQ	97.1 (2.2)	97.7 (3.7)	101.2 (0.7)	100.6 (3.7)	103.2 (5.9)	100.0 (3.5)	101.3 (1.4)	99.9 (1.9)	100.4 (2.0)	102.6 (4.1)	100.7 (0.3)	102.6 (1.2)
LQC	95.8 (4.7)	100.1 (4.8)	90.7 (1.4)	100.3 (3.0)	96.7 (7.2)	98.6 (6.6)	85.0 (3.7)	102.2 (5.8)	100.2 (6.6)	85.0 (3.7)	97.1 (1.5)	94.4 (2.3)
MQC	98.5 (4.1)	101.9 (5.3)	97.7 (0.4)	98.5 (2.4)	97.0 (6.2)	100.5 (3.4)	98.6 (1.8)	98.4 (4.0)	99.4 (1.6)	90.5 (2.7)	99.3 (0.4)	97.9 (0.8)
HQC	96.1 (3.5)	98.3 (6.7)	102.6 (0.8)	102.3 (3.8)	103.8 (3.8)	101.2 (4.0)	104.3 (1.0)	103.5 (2.5)	100.9 (3.5)	106.8 (2.3)	101.2 (0.6)	102.0 (1.6)
r^2^ value	0.9989652	0.998917	0.9989498	0.9997173	0.9986158	0.9997911	0.9994429	0.9995696	0.9998586	0.9985048	0.9998422	0.9992636
gradient	y = 0.86970x	y = 11.1922x	y = 0.22328x	y = 0.43934x	y = 0.48084x	y = 1.19070x	y = 0.24105x	y = 1.98693x	y = 1.11667x	y = 1.99402x	y = 1.18951x	y = 0.81954x
y-intercept	−0.000136	0.052039	−0.001203	−0.001630	−0.002647	0.003514	−0.001818	−0.009050	−0.001200	−0.045526	−0.000901	−0.004389
Between-run												
LLOQ	100.6 (5.1)	89.1 (4.0)	105.5 (1.9)	100.2 (9.1)	98.2 (7.5)	101.2 (9.9)	112.1 (2.9)	96.6 (6.3)	107.7 (10.9)	116.4 (1.3)	101.6 (3.0)	108.8 (2.5)
ULOQ	96.8 (1.9)	99.4 (4.9)	100.9 (1.3)	98.6 (2.6)	99.0 (3.2)	99.0 (2.3)	100.9 (0.9)	96.3 (3.9)	99.8 (1.7)	100.6 (1.5)	100.1 (0.3)	101.8 (1.3)
LQC	97.9 (4.7)	105.8 (5.3)	95.3 (2.1)	98.3 (2.4)	96.1 (3.0)	96.1 (3.5)	85.5 (1.1)	97.4 (4.6)	98.6 (6.8)	85.6 (5.9)	97.9 (0.9)	95.9 (1.9)
MQC	102.6 (3.5)	104.9 (2.8)	96.9 (1.3)	98.9 (1.6)	101.8 (3.5)	98.5 (3.9)	98.6 (0.8)	101.0 (2.6)	101.5 (1.3)	96.6 (4.1)	99.5 (0.4)	97.7 (1.4)
HQC	99.8 (5.9)	104.3 (3.5)	102.2 (0.3)	101.7 (2.3)	101.9 (5.3)	102.9 (2.4)	103.0 (0.4)	99.4 (3.4)	100.6 (2.2)	108.5 (4.4)	101.6 (0.3)	103.3 (1.0)
r^2^ value	0.9990416	0.9988099	0.9995495	0.9998553	0.9995754	0.9998414	0.9996996	0.9985859	0.9998911	0.9988101	0.999935	0.9995843
gradient	y = 0.74695x	y = 10.9393x	y = 0.16590x	y = 0.35829x	y = 0.46184x	y = 1.25148x	y = 0.20729x	y = 1.86809x	y = 0.79040x	y = 2.00391x	y = 1.25686x	y = 1.09679x
y-intercept	−0.000178	0.091889	−0.000263	−0.000033	0.000713	0.001230	−0.001247	0.008453	0.000991	−0.0390911	0.000574	−0.000973

Accuracy (%) is denoted by mean concentration over nominal concentration and is accepted if ±15% or ±20% (for LLOQ). LLOQ: Lower limit-of-quantification, ULOQ: Upper limit-of-quantification, LQC: Low QC, MQC:. Mid QC, HQC: High QC. CAZ: ceftazidime, FEP: cefepime, PIP: piperacillin, ATM: aztreonam, MEM: meropenem, DOR: doripenem, LVX: levofloxacin, CEFT: ceftolozane, TZB: tazobactam, TGC: tigecycline, AVI: avibactam, FOS: fosfomycin. Precision (%CV) is denoted in parenthesis.

**Table 4:** Clinical characteristics of the four patients on fosfomycin-based combination therapy for carbapenem-resistant bacterial infections.

Patient No.	Age (Years), Gender	Weight, Height	Type Of Carbapenem-Resistant Bacterial Infection	Renal Function Status	Antibiotic Combination Regimen
001	68, Male	48 kg, 168 cm	*Achromobacter xylosoxidans* hospital-associated pneumonia	Normal, not on dialysis	Fosfomycin 8 g every 8 h as a 2-h infusion + Piperacillin/tazobactam 4.5 g every 6 h as a 1-h infusion + Levofloxacin 750 mg every 24 h
002	65, Female	86.4 kg, 154 cm	*Acinetobacter baumannii* Tenckhoff catheter exit site infection	Impaired, on Intermittent hemodialysis	Fosfomycin 8 g on dialysis days (3 g to be administered at least 4 h before dialysis, and 5 g to be administered at the end of the dialysis session) + Aztreonam 2 g every 12 h as a 2-h infusion
003	74, Female	45.6 kg, 150 cm	*Acinetobacter baumannii* ventilator-associated pneumonia	Impaired, on Intermittent hemodialysis	Fosfomycin 8 g on dialysis days (3 g to be administered at least 4 h before dialysis, and 5 g to be administered at the end of the dialysis session) + Cefepime 1 g every 12 h as a 4-h infusion
004	37, Male	104 kg, 178 cm	*Pseudomonas aeruginosa* bilateral gluteal pressure ulcers	Normal, not on dialysis	Fosfomycin 8 g every 8 h as a 3-h infusion + Meropenem 2 g every 8 h as a 3-h infusion + Levofloxacin 500 mg every 12 h

**Table 5:** Accuracy and precision of plasma quality control samples tested within batches of study samples.

Compound	FEP	PIP	ATM	MEM	LVX	TZB	FOF
Accuracy (%)							
**Patient 001**							
LLOQ		117.3 (2.9)			114.4 (2.0)	107.3 (5.4)	101.8 (1.8)
ULOQ		101.4 (2.4)			98.9 (1.1)	100.6 (2.7)	99.9 (1.7)
LQC		102.7 (1.0)			99.4 (2.5)	111.0 (5.6)	106.9 (1.9)
MQC		91.8 (1.8)			99.7 (1.7)	98.2 (3.3)	97.8 (0.5)
HQC		100.0 (1.8)			103.0 (0.4)	108.1 (2.5)	103.2 (0.9)
r^2^ value		0.9987362			0.9986969	0.9994183	0.9997751
Linearity Equation		y = 0.28770x − 0.167125			y = 0.90712x − 0.011084	y = 0.97505x − 0.027416	y = 1.08956x − 0.005619
**Patient 002**							
LLOQ			92.7 (1.57)				104.0 (3.85)
ULOQ			98.3 (2.89)				103.2 (1.46)
LQC			98.5 (6.18)				98.7 (3.00)
MQC			105.1 (3.58)				102.8 (0.42)
HQC			102.5 (4.42)				102.8 (1.03)
r^2^ value			0.9989275				0.999259
Linearity Equation			y = 0.42009x + 0.002480				y = 0.74937x + 0.004796
**Patient 003**							
LLOQ	81.4 (1.68)						89.1 (0.49)
ULOQ	99.3 (4.27)						102.4 (1.45)
LQC	99.5 (7.56)						104.1 (0.97)
MQC	102.7 (4.37)						97.9 (1.76)
HQC	101.8 (1.81)						101.7 (0.60)
r^2^ value	0.9988506						0.9985925
Linearity Equation	y = 8.19150x + 0.060959						y = 0.81900x + 0.007561
**Patient 004**							
LLOQ				99.4 (5.43)	111.6 (2.90)		96.5 (2.23)
ULOQ				98.3 (0.60)	101.2 (0.84)		99.0 (0.58)
LQC				100.5 (2.11)	98.5 (1.50)		99.8 (2.57)
MQC				104.6 (1.67)	102.7 (0.69)		100.1 (1.48)
HQC				95.7 (3.95)	101.8 (0.75)		96.45 (1.39)
r^2^ value				0.9995982	0.9997402		0.9999429
Linearity Equation				y = 0.587167x − 0.000061	y = 0.297456x − 0.001252		y = 0.821999x + 0.006490

## Supplementary Materials

The following are available online at https://www.mdpi.com/article/10.3390/antibiotics11010054/s1, Figure S1: Aztreonam_IS (left) and aztreonam (right) against 6 different plasma sources and blank CAMHB, Figure S2: Avibactam_IS (left) and avibactam (right) against 6 different plasma sources and blank CAMHB, Figure S3: Ceftazidime_IS (left) and ceftazidime (right) against 6 different plasma sources and blank CAMHB, Figure S4: Ceftolozane_IS (left) and ceftolozane (right) against 6 different plasma sources and blank CAMHB, Figure S5: Doripenem_IS (left) and doripenem (right) against 6 different plasma sources and blank CAMHB, Figure S6: Cefepime_IS (left) and cefepime (right) against 6 different plasma sources and blank CAMHB, Figure S7: Fosfomycin_IS (left) and fosfomycin (right) against 6 different plasma sources and blank CAMHB, Figure S8: Levofloxacin_IS (left) and levofloxacin (right) against 6 different plasma sources and blank CAMHB, Figure S9: Meropenem_IS (left) and meropenem (right) against 6 different plasma sources and blank CAMHB, Figure S10: Piperacillin_IS (left) and piperacillin (right) against 6 different plasma sources and blank CAMHB, Figure S11: Tazobactam_IS (left) and tazobactam (right) against 6 different plasma sources and blank CAMHB, Figure S12: Tigecycline_IS (left) and tigecycline (right) against 6 different plasma sources and blank CAMHB, Table S1: Mass spectrometry parameters for the selected antibiotics and their internal standards, Table S2: Comparison of area of blanks after ULOQ injection to area of LLOQ for all analytes and their corresponding internal standards, Table S3: Computation of limit of detection (LOD) from observed signal to noise ratio (S/N) at lowest calibrator levels, Table S4: 32-day stability test of all antibiotic and internal standard stock solutions stored at −80 °C, Table S5: Matrix Factor and internal standard normalized matrix factor in plasma, Table S6: Matrix Factor and internal standard normalized matrix factor in CAMHB, Table S7: Stability of plasma extracted samples after 7 h in autosampler, Table S8: Stability of CAMHB extracted samples after 7 h in autosampler, Table S9: Stability of plasma extracted samples after 15 h in autosampler, Table S10: Stability of CAMHB extracted samples after 15 h in autosampler, Table S11: Stability after 2 weeks at −30 °C for drug combination plasma samples, Table S12: Stability after 4 weeks at −30 °C for drug combination plasma samples, Table S13: Stability after 2 weeks at −80 °C for drug combination plasma samples, Table S14: Stability after 4 weeks at −80 °C for drug combination plasma samples, Table S15: Stability after 2 weeks at −30 °C for drug combination CAMHB samples, Table S16: Stability after 4 weeks at −30 °C for drug combination CAMHB samples, Table S17: Stability after 2 weeks at −80 °C for drug combination CAMHB samples, Table S18: Stability after 4 weeks at −80 °C for drug combination CAMHB samples.

## References

[1] Versporten A., Zarb P., Caniaux I., Gros M.F., Drapier N., Miller M., Jarlier V., Nathwani D., Goossens H., Koraqi A. (2018). Antimicrobial Consumption and Resistance in Adult Hospital Inpatients in 53 Countries: Results of an Internet-Based Global Point Prevalence Survey. Lancet Glob. Health.

[2] Vardakas K.Z., Legakis N.J., Triarides N., Falagas M.E. (2016). Susceptibility of Contemporary Isolates to Fosfomycin: A Systematic Review of the Literature. Int. J. Antimicrob. Agents.

[3] Falagas M.E., Giannopoulou K.P., Kokolakis G.N., Rafailidis P.I. (2008). Fosfomycin: Use Beyond Urinary Tract and Gastrointestinal Infections. Clin. Infect. Dis..

[4] Walsh C.C., Landersdorfer C.B., McIntosh M.P., Peleg A.Y., Hirsch E.B., Kirkpatrick C.M., Bergen P.J. (2016). Clinically Relevant Concentrations of Fosfomycin Combined with Polymyxin B, Tobramycin or Ciprofloxacin Enhance Bacterial Killing of Pseudomonas Aeruginosa, but Do Not Suppress the Emergence of Fosfomycin Resistance. J. Antimicrob. Chemother..

[5] VanScoy B., McCauley J., Bhavnani S.M., Ellis-Grosse E.J., Ambrose P.G. (2016). Relationship between Fosfomycin Exposure and Amplification of Escherichia Coli Subpopulations with Reduced Susceptibility in a Hollow-Fiber Infection Model. Antimicrob. Agents Chemother..

[6] Cheng Y.-S., Williamson P.R., Zheng W. (2019). Improving Therapy of Severe Infections through Drug Repurposing of Synergistic Combinations. Curr. Opin. Pharmacol..

[7] Decosterd L.A., Widmer N., André P., Aouri M., Buclin T. (2016). The Emerging Role of Multiplex Tandem Mass Spectrometry Analysis for Therapeutic Drug Monitoring and Personalized Medicine. TrAC Trends Anal. Chem..

[8] Roberts J.A., Abdul-Aziz M.H., Lipman J., Mouton J.W., Vinks A.A., Felton T.W., Hope W.W., Farkas A., Neely M.N., Schentag J.J. (2014). Individualised Antibiotic Dosing for Patients Who Are Critically Ill: Challenges and Potential Solutions. Lancet Infect. Dis..

[9] Martens-Lobenhoffer J., Bode-Böger S.M. (2015). A Validated Method for the Quantification of Fosfomycin in Human Plasma by Liquid Chromatography–Tandem Mass Spectrometry. J. Chromatogr. B.

[10] Shopova T., Hüppe T., Wolf B., Sessler D.I., Volk T., Groesdonk H.V., Kreuer S., Maurer F. (2021). Quantitative Determination of Fosfomycin in 10 ΜL of Plasma and Dialysate by Hydrophilic Interaction Liquid Chromatography Electrospray Ionization Mass Spectrometry. J. Chromatogr. Sci..

[11] Wijma R.A., Bahmany S., Wilms E.B., van Gelder T., Mouton J.W., Koch B.C.P. (2017). A Fast and Sensitive LC–MS/MS Method for the Quantification of Fosfomycin in Human Urine and Plasma Using One Sample Preparation Method and HILIC Chromatography. J. Chromatogr. B Anal. Technol. Biomed. Life Sci..

[12] Parker S.L., Lipman J., Dimopoulos G., Roberts J.A., Wallis S.C. (2015). A Validated Method for the Quantification of Fosfomycin on Dried Plasma Spots by HPLC–MS/MS: Application to a Pilot Pharmacokinetic Study in Humans. J. Pharm. Biomed. Anal..

[13] Gandhi A., Matta M., Garimella N., Zere T., Weaver J. (2018). Development and Validation of a LC-MS/MS Method for Quantitation of Fosfomycin—Application to In Vitro Antimicrobial Resistance Study Using Hollow-Fiber Infection Model. Biomed. Chromatogr..

[14] Parker S.L., Lipman J., Roberts J.A., Wallis S.C. (2015). A Simple LC–MS/MS Method Using HILIC Chromatography for the Determination of Fosfomycin in Plasma and Urine: Application to a Pilot Pharmacokinetic Study in Humans. J. Pharm. Biomed. Anal..

[15] El-Najjar N., Jantsch J., Gessner A. (2017). A Rapid Liquid Chromatography-Tandem Mass Spectrometry for the Quantification of Fosfomycin in Plasma, Urine, and Aqueous Fluids. J. Chromatogr. B.

[16] Magréault S., Leroux S., Touati J., Storme T., Jacqz-Aigrain E. (2019). UPLC/MS/MS Assay for the Simultaneous Determination of Seven Antibiotics in Human Serum–Application to Pediatric Studies. J. Pharm. Biomed. Anal..

[17] Barco S., Mesini A., Barbagallo L., Maffia A., Tripodi G., Pea F., Saffioti C., Castagnola E., Cangemi G. (2020). A Liquid Chromatography-Tandem Mass Spectrometry Platform for the Routine Therapeutic Drug Monitoring of 14 Antibiotics: Application to Critically Ill Pediatric Patients. J. Pharm. Biomed. Anal..

[18] Decosterd L.A., Mercier T., Ternon B., Cruchon S., Guignard N., Lahrichi S., Pesse B., Rochat B., Burger R., Lamoth F. (2020). Validation and Clinical Application of a Multiplex High Performance Liquid Chromatography – Tandem Mass Spectrometry Assay for the Monitoring of Plasma Concentrations of 12 Antibiotics in Patients with Severe Bacterial Infections. J. Chromatogr. B.

[19] Cai Y., Chua N.G., Lim T.-P., Teo J.Q.-M., Lee W., Kurup A., Koh T.-H., Tan T.-T., Kwa A.L. (2016). From Bench-Top to Bedside: A Prospective In Vitro Antibiotic Combination Testing (IACT) Service to Guide the Selection of Rationally Optimized Antimicrobial Combinations against Extensively Drug Resistant (XDR) Gram Negative Bacteria (GNB). PLoS ONE.

[20] Food and Drug Administration (2018). Guidance for Industry: Bioanalytical Method Validation.

[21] Beta-Lactam Therapeutic Drug Monitoring in Singapore (BLAST-2). https://clinicaltrials.gov/ct2/show/NCT04450680.

[22] European Medicines Agency (2019). ICH Guideline M10 on Bioanalytical Method Validation Step 2b.

[23] Rigo-Bonnin R., Ribera A., Arbiol-Roca A., Cobo-Sacristán S., Padullés A., Murillo Ò., Shaw E., Granada R., Pérez-Fernández X.L., Tubau F. (2017). Development and Validation of a Measurement Procedure Based on Ultra-High Performance Liquid Chromatography-Tandem Mass Spectrometry for Simultaneous Measurement of β-Lactam Antibiotic Concentration in Human Plasma. Clin. Chim. Acta.

[24] Bruck F., Roberts M.S., Roberts J.A., Robertson T.A. (2014). Simultaneous Determination of Seven-Lactam Antibiotics in Human Plasma for Therapeutic Drug Monitoring and Pharmacokinetic Studies. J. Chromatogr. B.

[25] Ghimire S., van Hateren K., Vrubleuskaya N., Koster R., Touw D., Alffenaar J.-W.C. (2018). Determination of Levofloxacin in Human Serum Using Liquid Chromatography Tandem Mass Spectrometry. J. Appl. Bioanal..

[26] Naser F.J., Mahieu N.G., Wang L., Spalding J.L., Johnson S.L., Patti G.J. (2018). Two Complementary Reversed-Phase Separations for Comprehensive Coverage of the Semipolar and Nonpolar Metabolome. Anal. Bioanal. Chem..

[27] Forier K., Van Heck V., Carlier M., Van Braeckel E., Van Daele S., De Baets F., Schelstraete P., Haerynck F., Stove V., Van Simaey L. (2018). Development and Validation of an LC Tandem MS Assay for the Quantification of β-Lactam Antibiotics in the Sputum of Cystic Fibrosis Patients. J. Antimicrob. Chemother..

[28] Avery L.M., Sutherland C.A., Nicolau D.P. (2019). In Vitro Investigation of Synergy among Fosfomycin and Parenteral Antimicrobials against Carbapenemase-Producing Enterobacteriaceae. Diagn. Microbiol. Infect. Dis..

[29] Asuphon O., Montakantikul P., Houngsaitong J., Kiratisin P., Sonthisombat P. (2016). Optimizing Intravenous Fosfomycin Dosing in Combination with Carbapenems for Treatment of Pseudomonas Aeruginosa Infections in Critically Ill Patients Based on Pharmacokinetic/Pharmacodynamic (PK/PD) Simulation. Int. J. Infect. Dis..

[30] Cuba G.T., Rocha-Santos G., Cayô R., Streling A.P., Nodari C.S., Gales A.C., Pignatari A.C.C., Nicolau D.P., Kiffer C.R. (2020). V In Vitro Synergy of Ceftolozane/Tazobactam in Combination with Fosfomycin or Aztreonam against MDR Pseudomonas Aeruginosa. J. Antimicrob. Chemother..

[31] Perdigão Neto L.V., Oliveira M.S., Martins R.C.R., Marchi A.P., Gaudereto J.J., Da Costa L.A.T.J., De Lima L.F.A., Takeda C.F.V., Costa S.F., Levin A.S. (2019). Fosfomycin in Severe Infections Due to Genetically Distinct Pan-Drug-Resistant Gram-Negative Microorganisms: Synergy with Meropenem. J. Antimicrob. Chemother..

[32] Mikhail S., Singh N.B., Kebriaei R., Rice S.A., Stamper K.C., Castanheira M., Rybak M.J. (2019). Evaluation of the Synergy of Ceftazidime-Avibactam in Klebsiella Pneumoniae and Pseudomonas Aeruginosa. Antimicrob. Agents Chemother..

[33] Papp-Wallace K.M., Zeiser E.T., Becka S.A., Park S., Wilson B.M., Winkler M.L., D’Souza R., Singh I., Sutton G., Fouts D.E. (2020). Ceftazidime-Avibactam in Combination with Fosfomycin: A Novel Therapeutic Strategy against Multidrug-Resistant Pseudomonas Aeruginosa. J. Infect. Dis..

[34] Magiorakos A.-P., Srinivasan A., Carey R.B., Carmeli Y., Falagas M.E., Giske C.G., Harbarth S., Hindler J.F., Kahlmeter G., Olsson-Liljequist B. (2012). Multidrug-Resistant, Extensively Drug-Resistant and Pandrug-Resistant Bacteria: An International Expert Proposal for Interim Standard Definitions for Acquired Resistance. Clin. Microbiol. Infect..

[35] Schmidt J.J., Bode-Böger S.M., Wilhelmi M., Omar M., Martens-Lobenhoffer J., Welte T., Kielstein J.T. (2016). Pharmacokinetics and Total Removal of Fosfomycin in Two Patients Undergoing Intermittent Haemodialysis and Extended Dialysis: Prescription Needs to Avoid under-Dosing. J. Antimicrob. Chemother..

