# Supplementary material for: Quantification of Fosfomycin in Combination with Nine Antibiotics in Human Plasma and Cation-Adjusted Mueller-Hinton II Broth via LCMS
Source: Antibiotics (Basel). 2022 Jan 2;11(1):54. doi: 10.3390/antibiotics11010054 (PMC8772704; doi:10.3390/antibiotics11010054)
Supplement: Supplementary file 1 [file antibiotics-11-00054-s001.zip › antibiotics-1519569-supplementary.pdf]

Supplementary Data

Specificity and Selectivity

No interfering compounds were detected on blank plasma from 6 different sources or blank CAMHB sample. Spiking of blank samples with stable isotope internal standards confirmed no presence of the unlabelled standards. Similarly, when spiking plasma or CAMHB with antibiotics at the highest concentration, no peaks were observed in the chromatograms of the corresponding stable-isotope internal standards.

Figure S1. Aztreonam_IS (left) and aztreonam (right) against 6 different plasma sources and blank CAMHB

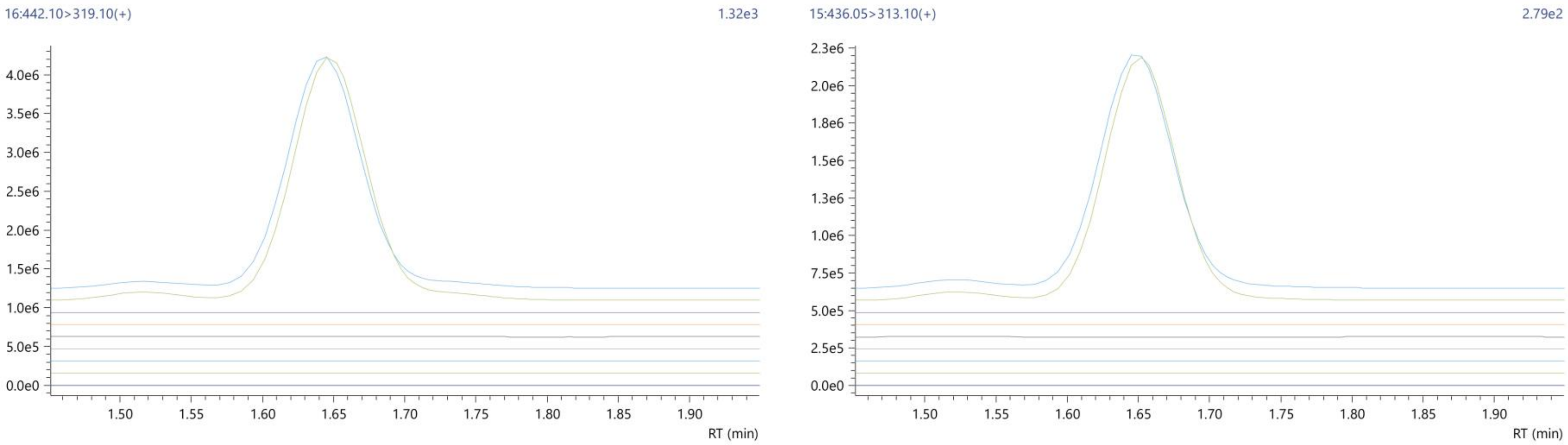

Figure S2. Avibactam_IS (left) and avibactam (right) against 6 different plasma sources and blank CAMHB

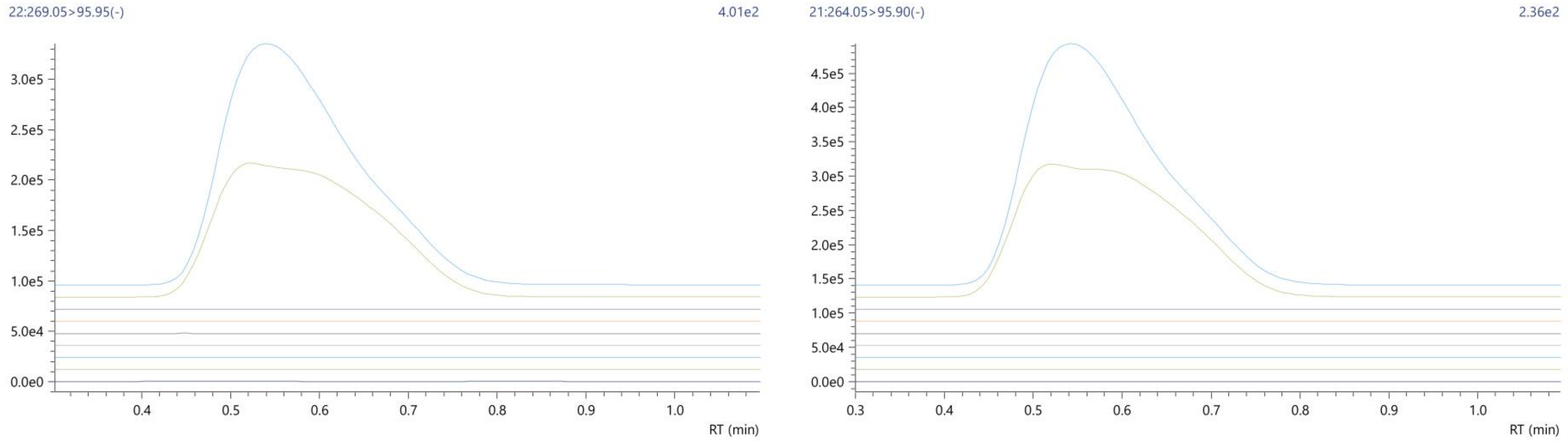

Figure S3. Ceftazidime_IS (left) and ceftazidime (right) against 6 different plasma sources and blank CAMHB

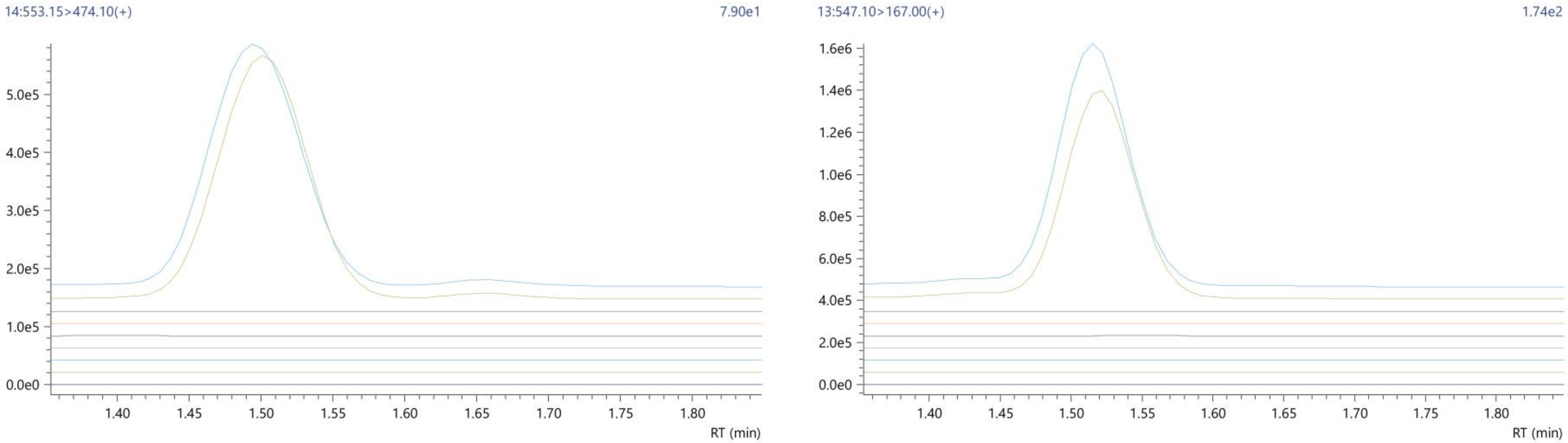

Figure S4. Ceftolozane_IS (left) and ceftolozane (right) against 6 different plasma sources and blank CAMHB

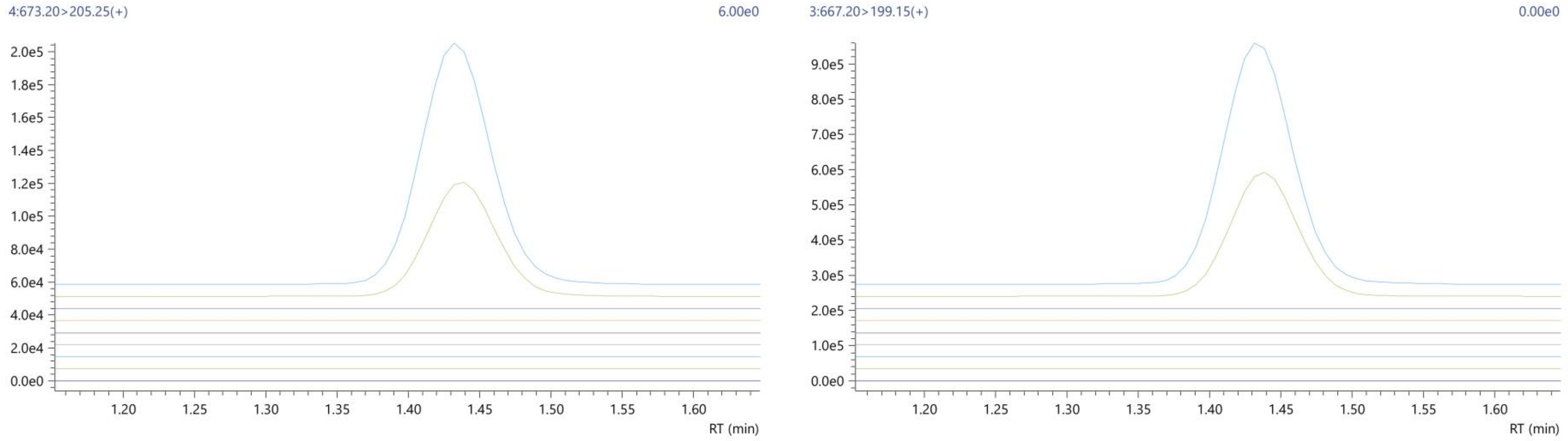

Figure S5. Doripenem_IS (left) and doripenem (right) against 6 different plasma sources and blank CAMHB

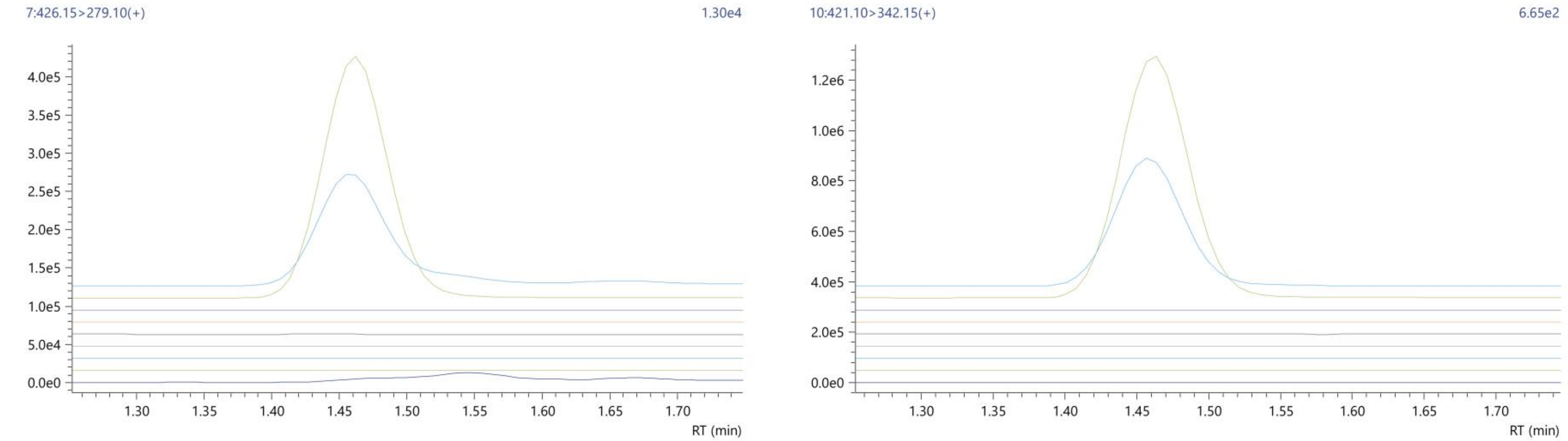

Figure S6. Cefepime_IS (left) and cefepime (right) against 6 different plasma sources and blank CAMHB

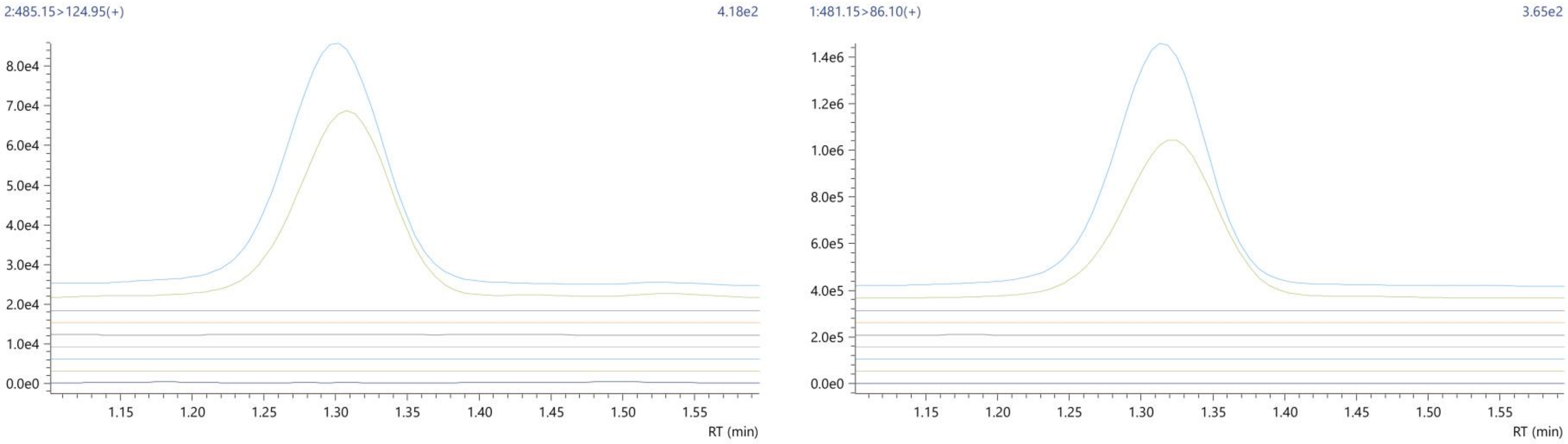

Figure S7. Fosfomycin_IS (left) and fosfomycin (right) against 6 different plasma sources and blank CAMHB

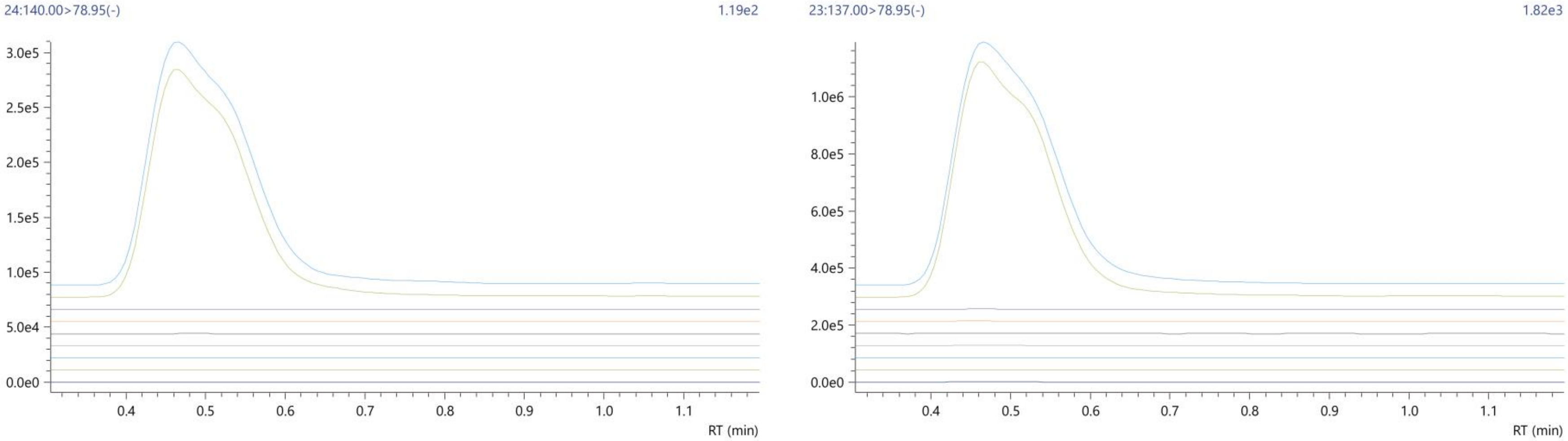

Figure S8. Levofloxacin_IS (left) and levofloxacin (right) against 6 different plasma sources and blank CAMHB

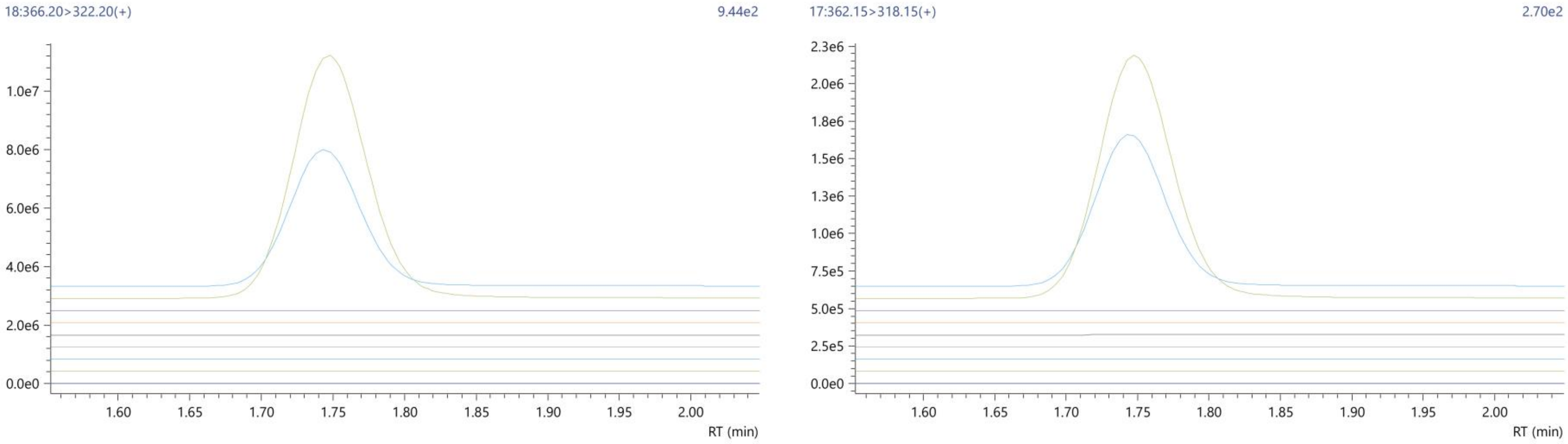

Figure S9. Meropenem_IS (left) and meropenem (right) against 6 different plasma sources and blank CAMHB

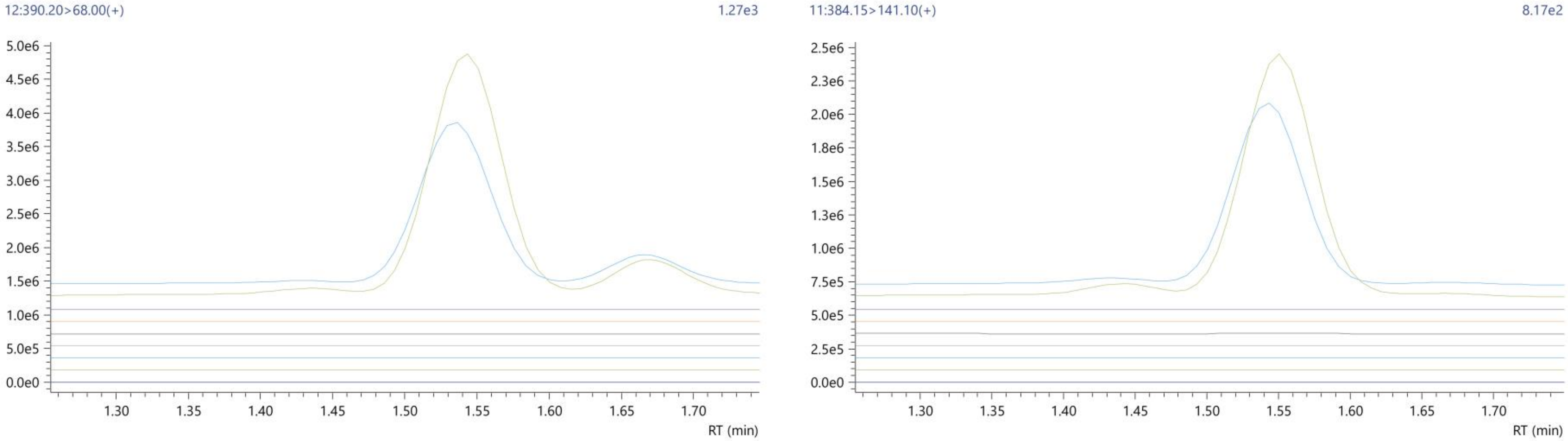

Figure S10. Piperacillin_IS (left) and piperacillin (right) against 6 different plasma sources and blank CAMHB

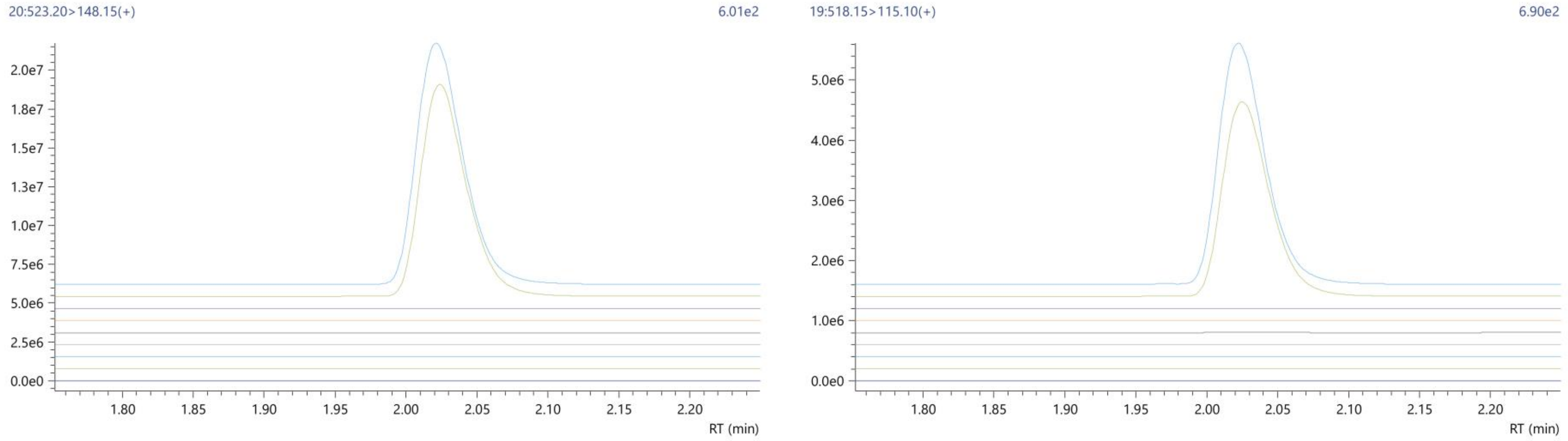

Figure S11. Tazobactam_IS (left) and tazobactam (right) against 6 different plasma sources and blank CAMHB

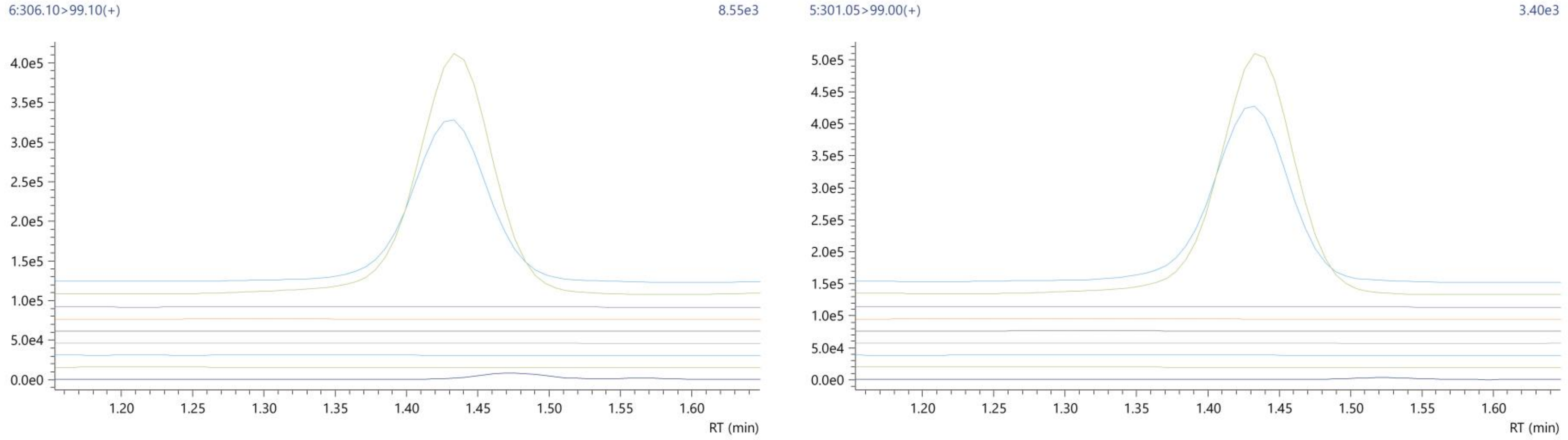

Figure S12. Tigecycline_IS (left) and tigecycline (right) against 6 different plasma sources and blank CAMHB

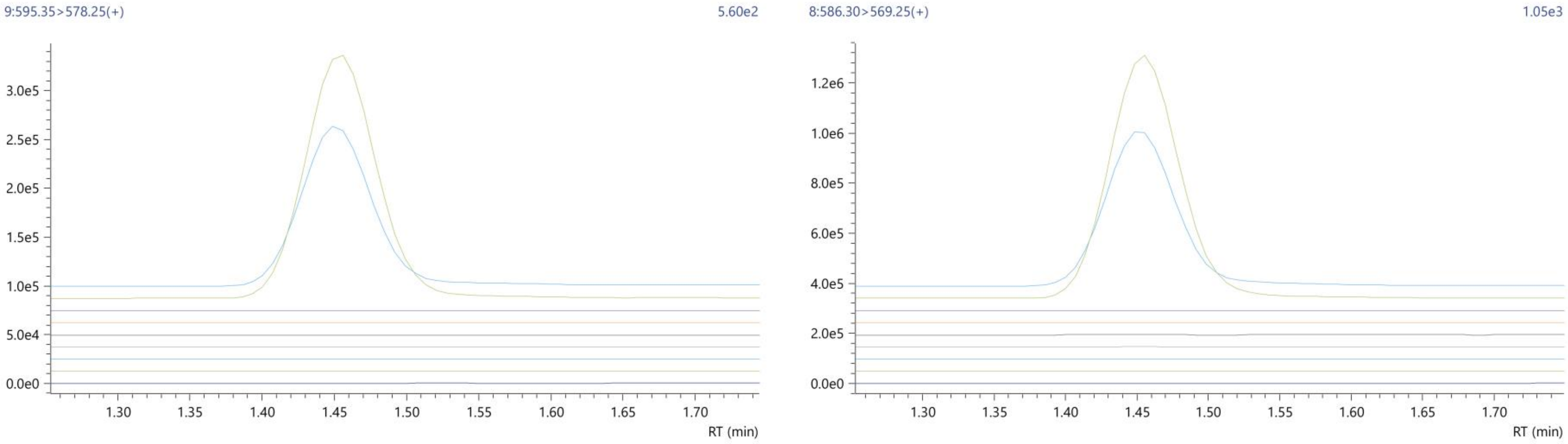

Liquid chromatography conditions

The following 4.5 min gradient profile was used: The elution started at 0-0.7 min with 3% B, then linearly increased to 20% B until 0.8 min, followed by an increase to 90% B by 1.5 min and keeping constant till 2.5 min. Composition of B then decreased to its initial condition of 3% in 0.5 min and kept constant for the remaining 1.5 min. Total flow linearly increased from 0.3 to 0.6 mL/min at 0.9-1.0 min and keeping constant for 0.5 min, before linearly decreased to 0.3 mL/min from 1.5-1.6 min. The LC flow was directed into the MS detector between 0.3 and 3.5 min using the high-pressure switching valve. The total analysis time was 4.5 min. The injection volume was 10 µL. Column and autosampler temperature were set at 40 °C and 4 °C respectively.

Table S1. Mass spectrometry parameters for the selected antibiotics and their internal standards

Code	Antibiotic	ESI mode	Retention time (min)	Quantification transition	Quantification energies[a,b,c]	Confirmation transition	Confirmation energies[a,b,c]	Dwell (msec)
FOF	Fosfomycin	-	0.48	137.00 > 78.95	(25,14,15)	137.00 > 62.95	(15,14,13)	10
FOF_IS	$[^{13}C_3]$-Fosfomycin	-	0.48	140.00 > 78.95	(27,14,16)	140.00 > 63.00	(16,14,12)	10
AVI	Avibactam	-	0.55	264.05 > 95.90	(24,30,19)	264.05 > 79.95	(28,30,16)	50
AVI_IS	$[^{13}C_5]$-Avibactam	-	0.55	269.05 > 95.95	(27,30,19)	269.05 > 79.90	(26,29,15)	50
FEP	Cefepime	+	1.25	481.15 > 86.10	(-14,-17,-14)	481.15 > 395.95	(-13,-25,-26)	10
FEP_IS	$[^{13}C, ^{2}H_3]$-Cefepime	+	1.24	485.15 > 124.95	(-54,-29,-22)	485.15 > 276.90	(-23,-29,-30)	10
TZB	Tazobactam	+	1.39	301.05 > 99.00	(-26,-16,-16)	301.05 > 94.10	(-27,-20,-17)	10
TZB_IS	$[^{13}C_2, ^{15}N_3]$-Tazobactam	+	1.39	306.10 > 99.10	(-27,-21,-19)	306.10 > 168.10	(-14,-21,-16)	10
CEFT	Ceftolozane	+	1.41	667.20 > 199.15	(-15,-24,-21)	667.20 > 139.20	(-30,-36,-22)	10
CEFT_IS	$[^{15}N_2, ^{2}H_4]$-Ceftolozane	+	1.41	673.20 >205.25	(-16,-36,-22)	673.20 > 138.90	(-34,-24,-26)	10
TGC	Tigecycline	+	1.44	586.30 > 569.25	(-22,-38,-28)	586.30 > 513.25	(-26,-38,-24)	10
TGC_IS	$[^{2}H_9]$-Tigecycline	+	1.44	595.35 > 578.25	(-22,-40,-28)	595.35 > 514.35	(-28,-36,-36)	10
DOR	Doripenem	+	1.45	421.10 > 342.15	(-15,-22,-15)	421.10 > 112.10	(-42,-13,-10)	10
DOR_IS	$[^{2}H_5]$-Doripenem	+	1.45	426.15 > 279.10	(-19,-27,-18)	426.15 > 347.15	(-14,-25,-23)	10
CAZ	Ceftazidime	+	1.49	547.10 > 167.00	(-25,-20,-10)	547.10 > 396.30	(-18,-20,-26)	10
CAZ_IS	$[^{2}H_6]$-Ceftazidime	+	1.48	553.15 > 474.10	(-16,-20,-12)	553.15 > 125.80	(-39,-20,-22)	10
MEM	Meropenem	+	1.52	384.15 > 141.10	(-27,-25,-13)	384.15 > 340.15	(-17,-12,-11)	2
MEM_IS	$[^{2}H_6]$-Meropenem	+	1.51	390.20 > 68.00	(-40,-26,-25)	390.20 > 147.15	(-17,-25,-14)	2
ATM	Aztreonam	+	1.62	436.05 > 313.10	(-26,-28,-21)	436.05 >356.00	(-11,-23,-16)	2

ATM_IS	[$^{2}H_{6}$]-Aztre-onam	+	1.61	442.10 > 319.10	(-16,-29,-21)	442.10 > 362.15	(-11,-30,-16)	2
LVX	Levofloxa-cin	+	1.74	362.15 > 318.15	(-29,-19,-14)	362.15 > 261.15	(-17,-19,-26)	10
LVX_IS	[^{13}C, $^{2}H_{3}$]-Levoflexa-cin	+	1.73	366.20 > 322.20	(-19,-24,-21)	366.20 > 261.10	(-29,-24,-26)	10
PIP	Piperacillin	+	2.02	518.15 > 115.10	(-50,-28,-19)	518.15 > 160.00	(-10,-28,-27)	2
PIP_IS	[$^{2}H_{5}$]-Pipe-racillin	+	2.02	523.20 > 148.15	(-21,-28,-14)	523.20 > 160.05	(-15,-28,-27)	2

[a] Collision energies (eV), [b] Q1 pre bias (V) and [c] Q3 pre bias (V) are given in parenthesis.

Individual standard solutions (0.5 µg/mL) of each analyte and internal standard were directly injected to perform optimisation of their MS parameters (collision energy, Q1 pre bias and Q3 pre bias) to maximise the signal intensity of their multiple reaction monitoring (MRM) mass transitions.

energy, Q1 pre bias and Q3 pre bias) to maximise the signal intensity of their multiple reaction monitoring (MRM) mass transitions. Nitrogen was used as the nebulizing gas, with interface setting as follows: nebulizing gas flow of 3 L/min, heating gas flow of 15 L/min, drying gas flow of 5 L/min, interface temperature of 300°C, desolvation temperature of 526°C, desolvation line temperature of 250°C and heat block temperature of 200°C. The collision gas used was argon. The specific MS parameters for each antibiotic and their IS are shown in Table 2.

nebulizing gas, with interface setting as follows: nebulizing gas flow of 3 L/min, heating gas flow of 15 L/min, drying gas flow of 5 L/min, interface temperature of
300°C, desolvation temperature of 526°C, desolvation line temperature of 250°C and heat block temperature of 200°C. The collision gas used was argon.

Carryover

For all analytes and their corresponding stable-isotope internal standards, carryover was determined by calculating the area at the indicated retention times in the blank samples after injection of the ULOQ sample. No significant carry-over was present since all the peak areas of the analyte and internal standard in the blank sample were less than 20% of the area of the LLOQ or ,5% of the area of the internal standard, respectively.

Table S2. Comparison of area of blanks after ULOQ injection to area of LLOQ for all analytes and their corresponding internal standards

	Blank wash after Cal 8 in Plasma/CAMHB Analyte (Area)	ABX Signal intensity of Cal 1 LLOQ analyte (Average Area)	Carry-over analyte (%)	Blank wash after Cal 8 in Plasma/CAMHB IS (Area)	ABX_IS Signal intensity of Cal 1 IS (Average Area)	Carry-over IS (%)
**CAZ**	0	32,196	0.00	0	1,473,524	0.00
**FEP**	498	40,911	1.22	198	231,483	0.09
**PIP**	12,358	85,229	14.50	8375	36,080,689	0.02
**ATM**	400	57,858	0.69	1335	12,472,036	0.01
**MEM**	0	73,183	0.00	0	14,467,153	0.00
**DOR**	0	42,840	0.00	0	1,216,525	0.00
**LVX**	6155	46,911	13.12	5,076	37,978,729	0.01
**CEFT**	72	12,631	0.57	0	197,200	0.00
**TAZ**	475	15,709	3.02	1994	1,320,958	0.15
**TGC**	573	23,619	2.43	0	785,650	0.00
**AVI**	4104	31,452	13.05	1582	1,774,620	0.09
**FOF**	4268	88,691	4.81	1970	1,824,828	0.11

Table S3. Computation of limit of detection (LOD) from observed signal to noise ratio (S/N) at lowest calibrator levels

Compound	Nominal Concentration (mg/L)	MHB		Plasma	
		Signal to Noise Ratio	LOD (mg/L)	Signal to Noise Ratio	LOD (mg/L)
**CAZ**	0.6	246.44	0.00730	687.64	0.00262
**FEP**	0.3	884.22	0.00102	11285.53	0.00008
**PIP**	0.6	225.94	0.00797	217.81	0.00826
**ATM**	0.6	275.16	0.00654	317.13	0.00568
**MEM**	0.6	56.94	0.03161	287.23	0.00627
**DOR**	0.3	80.60	0.01117	225.01	0.00400
**LVX**	0.06	34.97	0.00515	84.61	0.00213
**CEFT**	0.6	5044.24	0.00036	Infinite	N/A
**TZB**	0.15	23.81	0.01890	58.01	0.00776
**TGC**	0.15	11.70	0.03848	43.70	0.01030
**AVI**	0.15	114.04	0.00395	201.58	0.00223
**FOF**	3	345.35	0.02606	219.59	0.04098

Stability of stock solutions

All stock solutions of antibiotics and their corresponding stable-isotope variants were measured prior to cold storage at -80 °C and tested at 32 days later. Stability was determined by comparing the peak area of each drug between its Day 1 and Day 32 measurement. Acceptable criteria for stability was met if the peak area at Day 32 does not exceed ±15% of the peak area at Day 1.

Table S4. 32-day stability test of all antibiotic and internal standard stock solutions stored at -80 °C

Date	**CAZ**	**FEP**	**PIP**	**ATM**	**MEM**	**DOR**	**LVX**	**CEFT**	**TZB**	**TGC**	**AVI**	**FOF**
Day 1	10,431,235	6,615,582	19,492,412	16,004,547	22,023,799	12,535,655	14,890,951	13,439,223	3,037,023	13,524,071	11,541,795	75,111,692
Day 32	10,611,444	7,178,822	19,586,244	15,963,742	22,129,378	12,425,998	14,655,449	14,472,636	2,825,529	13,871,512	11,845,345	71,953,862
32 Day Stability (%)	101.73	108.51	100.48	99.75	100.48	99.13	98.42	107.69	93.04	102.57	102.63	95.80
Date	**CAZ_IS**	**FEP_IS**	**PIP_IS**	**ATM_IS**	**MEM_IS**	**DOR_IS**	**LVX_IS**	**CEFT_IS**	**TZB_IS**	**TGC_IS**	**AVI_IS**	**FOF_IS**
Day 1	501,719	80,041	11,945,494	3,734,724	6,303,433	550,070	7,757,777	127,079	484,312	15,683	1,304,296	2,840,633
Day 32	490,297	76,982	11,733,051	3,658,164	6,983,876	507,631	8,045,178	115,774	475,074	14,254	1,331,943	2,851,512
32 Day Stability (%)	97.72	96.18	98.22	97.95	110.79	92.28	103.70	91.10	98.09	90.89	102.12	100.38

Table S5. Matrix Factor and internal standard normalized matrix factor in plasma

Compound	Level	Concentration (mg/L)	Average MF (%)	IS MF (%)	IS normalised MF (%)	(%) CV	Average recovery (%)	IS Recovery (%)	(%) CV
Ceftazidime	LQC	5	110.2	108.3	101.8	4.5	90.2	86.2	3.2
	MQC	150	108.3	108.1	100.2	3.9	86.8	86.6	3.2
	HQC	350	103.1	105.6	97.7	2.8	87.6	86.7	2.3
Cefepime	LQC	2.5	81.7	84.0	97.3	3.7	101.2	96.8	1.6
	MQC	75	100.8	95.5	105.5	5.2	92.0	95.5	6.6
	HQC	175	104.2	99.3	104.9	4.5	94.4	96.9	4.5
Piperacillin	LQC	5	93.4	94.6	98.7	2.3	97.6	95.0	2.6
	MQC	150	93.4	92.4	101.2	1.2	93.5	97.5	4.1
	HQC	350	94.0	92.9	101.1	1.9	92.3	97.5	3.8
Aztreonam	LQC	5	96.7	96.7	100.1	2.9	92.1	87.7	2.9
	MQC	150	96.9	96.1	100.8	0.8	89.6	91.7	2.6
	HQC	350	97.1	95.7	101.5	1.4	89.8	91.0	3.0
Meropenem	LQC	5	70.7	66.9	105.7	3.0	80.4	79.4	3.6
	MQC	150	72.2	68.4	105.5	1.8	82.5	86.4	3.6
	HQC	350	76.4	69.3	110.3	3.4	85.0	87.8	2.4
Doripenem	LQC	2.5	66.7	66.3	100.7	1.5	82.3	79.5	1.2
	MQC	75	64.7	64.7	99.9	2.0	79.7	83.9	5.9
	HQC	175	64.1	62.9	102.0	4.4	82.4	87.6	3.8
Levofloxacin	LQC	0.5	138.7	122.5	113.3	2.2	99.1	105.3	2.7
	MQC	15	117.0	111.0	105.4	4.1	92.3	103.8	5.3
	HQC	35	107.2	105.5	101.6	2.8	91.9	100.3	3.7
Ceftolozane	LQC	5	93.0	95.9	97.0	3.6	55.7	50.5	2.8
	MQC	150	96.7	96.8	99.9	1.9	45.8	45.7	4.2
	HQC	350	96.4	92.9	103.8	5.8	53.7	47.2	2.8
Tazobactam	LQC	1.25	103.1	102.7	100.4	2.2	93.7	94.8	5.3
	MQC	37.5	104.3	103.0	101.2	1.0	94.7	100.5	5.7

	HQC	87.5	102.7	101.4	101.2	1.7	96.8	103.9	3.0
Tigecycline	LQC	1.25	224.5	206.6	108.7	4.6	99.1	88.1	5.9
	MQC	37.5	145.5	138.7	105.0	1.8	90.3	92.3	1.2
	HQC	87.5	125.4	135.1	92.9	5.9	96.0	94.0	2.3
Avibactam	LQC	1.25	73.8	73.0	101.1	1.6	101.9	97.8	2.4
	MQC	37.5	77.0	78.8	97.6	1.6	95.9	98.1	3.4
	HQC	87.5	80.7	82.5	97.9	1.8	96.4	100.5	3.2
Fosfomycin	LQC	25	22.0	21.2	103.8	2.9	74.3	72.5	3.9
	MQC	750	25.4	28.0	90.7	2.3	70.3	73.2	3.8
	HQC	1750	36.5	34.2	106.9	4.0	72.1	76.7	3.8

Table S6. Matrix Factor and internal standard normalized matrix factor in CAMHB

Compound	Level	Concentration (mg/L)	Average MF (%)	IS MF (%)	IS normalised MF (%)	(%) CV	Average recovery (%)	Recovery IS (%)	(%) CV
Ceftazidime	LQC	5	129.2	112.6	114.7	1.3	98.2	97.1	2.8
	MQC	150	109.0	95.9	113.6	0.2	96.0	97.0	2.8
	HQC	350	96.0	89.0	107.8	4.0	97.4	95.2	3.3
Cefepime	LQC	2.5	105.0	105.9	99.1	2.8	99.4	99.5	5.3
	MQC	75	106.5	107.2	99.4	4.2	105.9	104.2	6.0
	HQC	175	120.2	119.6	100.5	3.6	90.9	91.5	5.2
Piperacillin	LQC	5	117.2	114.5	102.3	1.6	99.1	99.0	1.2
	MQC	150	108.6	108.6	100.1	2.6	98.5	100.0	1.4
	HQC	350	103.8	105.0	98.9	5.7	97.6	99.5	3.3
Aztreonam	LQC	5	87.7	87.9	99.8	2.0	96.2	96.4	2.1
	MQC	150	82.5	82.5	100.1	3.6	95.1	96.5	1.2
	HQC	350	80.5	81.7	98.5	4.8	94.6	95.9	3.9
Meropenem	LQC	5	65.1	61.5	105.7	2.5	85.2	87.8	1.5
	MQC	150	66.2	63.8	103.8	3.5	91.3	94.4	2.0

	HQC	350	64.6	61.5	105.0	4.3	92.6	94.8	3.1
Doripenem	LQC	2.5	36.9	41.1	89.9	1.4	88.6	89.2	3.1
	MQC	75	42.8	47.0	91.0	1.5	89.8	92.8	1.5
	HQC	175	43.2	48.3	89.5	4.3	90.9	94.1	4.7
Levofloxa-cin	LQC	0.5	64.8	58.4	111.0	1.0	100.0	107.0	3.2
	MQC	15	66.3	62.1	106.8	3.8	97.6	106.7	1.9
	HQC	35	64.5	64.5	100.0	3.8	96.5	100.4	3.1
Ceftolozane	LQC	5	102.0	103.1	99.0	3.2	98.7	98.6	1.7
	MQC	150	75.3	76.4	98.6	2.0	102.2	103.9	0.2
	HQC	350	81.7	78.7	103.8	6.1	96.6	98.4	2.7
Tazobactam	LQC	1.25	72.7	75.5	96.2	3.3	91.9	93.5	3.5
	MQC	37.5	85.1	85.9	99.0	0.5	87.7	88.0	1.5
	HQC	87.5	91.5	91.7	99.8	3.9	93.7	95.6	3.3
Tigecycline	LQC	1.25	226.2	216.7	104.4	4.8	87.2	89.8	1.0
	MQC	37.5	117.5	112.8	104.2	2.4	91.6	92.8	4.2
	HQC	87.5	110.2	111.4	98.9	1.9	95.3	94.5	2.0
Avibactam	LQC	1.25	114.7	115.8	99.1	0.6	104.0	104.8	0.4
	MQC	37.5	99.6	103.1	96.5	2.4	104.1	105.6	1.2
	HQC	87.5	89.4	94.2	95.0	5.6	102.1	104.0	3.3
Fosfomycin	LQC	25	17.6	17.4	101.2	1.7	99.9	98.9	1.0
	MQC	750	19.3	21.6	89.3	1.9	99.0	101.1	1.5
	HQC	1750	25.4	27.7	91.5	4.4	98.1	100.6	2.9

Autosampler stability at 4 °C (7h)

Table S7. Stability of plasma extracted samples after 7h in autosampler

Compound	Level	Nominal Concentration (mg/L)	Measured Concentration (mg/L)	(%) CV	Mean accuracy (%)	Deviation from nominal (%)
Ceftazidime	LQC	1	1.0	6.9	94.9	-5.1
	MQC	75	84.0	1.7	112.0	12.0
	HQC	150	164.5	3.4	109.6	9.6
Cefepime	LQC	1	0.9	6.0	94.2	-5.8
	MQC	100	101.1	1.1	101.1	1.1
	HQC	200	172.9	11.3	86.5	-13.5
Piperacillin	LQC	1	1.1	1.0	109.5	9.5
	MQC	150	126.0	0.4	84.0[a]	-16.0
	HQC	250	218.8	1.1	87.5	-12.5
Aztreonam	LQC	1	1.0	6.8	100.8	0.8
	MQC	150	151.8	2.8	101.2	1.2
	HQC	300	327.2	0.5	109.1	9.1
Meropenem	LQC	1	1.1	3.5	110.0	10.0
	MQC	100	95.5	4.6	95.5	-4.5
	HQC	200	199.1	0.6	99.6	-0.4
Doripenem	LQC	1	1.0	2.2	104.0	4.0
	MQC	87.5	88.1	2.5	100.7	0.7
	HQC	175	173.1	4.0	98.9	-1.1
Levofloxacin	LQC	0.5	0.6	1.4	110.7	10.7
	MQC	15	15.8	1.4	105.2	5.2
	HQC	30	30.4	0.5	101.3	1.3
Ceftolozane	LQC	1	1.1	8.9	106.0	6.0
	MQC	125	121.5	2.4	97.2	-2.8

	HQC	250	262.8	2.7	105.1	5.1
Tazobactam	LQC	1	1.1	0.9	113.6	13.6
	MQC	43.75	45.6	1.9	104.1	4.1
	HQC	87.5	97.4	0.9	111.3	11.3
Tigecycline	LQC	1	1.1	4.8	106.7	6.7
	MQC	10	11.3	3.2	112.7	12.7
	HQC	20	22.0	2.7	110.0	10.0
Avibactam	LQC	0.5	0.5	1.5	105.4	5.4
	MQC	15	15.1	0.5	100.7	0.7
	HQC	30	28.9	0.3	96.2	-3.8
Fosfomycin	LQC	10	10.5	2.7	104.5	4.5
	MQC	500	505.2	0.9	101.0	1.0
	HQC	1000	1029.0	1.4	102.9	2.9

[a]Value does not meet acceptance criteria for stability according to FDA guideline

Autosampler stability at 4 °C (7h)

Table S8. Stability of CAMHB extracted samples after 7h in autosampler

Compound	Level	Nominal Concentration (mg/L)	Measured Concentration (mg/L)	(%) CV	Mean accuracy (%)	Deviation from nominal (%)
Ceftazidime	LQC	1	1.1	5.0	110.0	10.0
	MQC	75	81.8	4.3	109.0	9.0
	HQC	150	163.8	3.2	109.2	9.2
Cefepime	LQC	1	1.1	1.3	112.0	12.0
	MQC	100	103.5	6.9	103.5	3.5
	HQC	200	193.4	4.6	96.7	-3.3
Piperacillin	LQC	1	1.1	0.7	113.7	13.7
	MQC	150	130.1	0.6	86.7	-13.3
	HQC	250	224.1	0.8	89.6	-10.4
Aztreonam	LQC	1	1.1	7.1	109.7	9.7
	MQC	150	161.2	4.5	107.5	7.5
	HQC	300	297.9	5.8	99.3	-0.7
Meropenem	LQC	1	1.0	1.4	104.0	4.0
	MQC	100	102.6	1.8	102.6	2.6
	HQC	200	215.4	3.9	107.7	7.7
Doripenem	LQC	1	1.1	3.5	104.8	4.8
	MQC	87.5	92.1	2.7	105.2	5.2
	HQC	175	180.4	1.6	103.1	3.1
Levofloxacin	LQC	0.5	0.6	0.3	113.0	13.0
	MQC	15	15.5	1.1	103.4	3.4
	HQC	30	29.6	1.3	98.8	-1.2
Ceftolozane	LQC	1	1.1	3.6	106.4	6.4

	MQC	125	137.3	0.4	109.8	9.8
	HQC	250	283.3	0.5	113.3	13.3
Tazobactam	LQC	1	1.1	5.0	109.4	9.4
	MQC	43.75	47.0	1.9	107.5	7.5
	HQC	87.5	95.9	2.6	109.6	9.6
Tigecycline	LQC	1	1.0	2.3	100.6	0.6
	MQC	10	10.3	2.5	103.1	3.1
	HQC	20	20.7	1.5	103.5	3.5
Avibactam	LQC	0.5	0.5	1.0	108.8	8.8
	MQC	15	15.6	0.8	104.2	4.2
	HQC	30	31.9	0.3	106.4	6.4
Fosfomycin	LQC	10	10.7	2.7	107.1	7.1
	MQC	500	479.4	1.7	95.9	-4.1
	HQC	1000	960.3	1.1	96.0	-4.0

Autosampler stability at 4 °C (15h)

Table S9. Stability of plasma extracted samples after 15h in autosampler

Compound	Level	Nominal Concentration (mg/L)	Measured Concentration (mg/L)	(%) CV	Mean accuracy (%)	Deviation from nominal (%)
Ceftazidime	LQC	1	0.9	6.5	93.8	-6.2
	MQC	75	83.6	2.1	111.5	11.5
	HQC	150	169.7	1.1	113.1	13.1
Cefepime	LQC	1	1.0	4.7	94.5	-5.5
	MQC	100	90.5	5.0	90.5	-9.5
	HQC	200	213.5	4.5	106.7	6.7
Piperacillin	LQC	1	1.1	1.2	109.9	9.9
	MQC	150	130.2	0.9	86.8	-13.2
	HQC	250	223.9	2.3	89.6	-10.4
Aztreonam	LQC	1	1.1	3.1	105.8	5.8
	MQC	150	153.7	0.9	102.4	2.4
	HQC	300	323.6	3.2	107.9	7.9
Meropenem	LQC	1	1.1	0.8	109.1	9.1
	MQC	100	98.9	2.1	98.9	-1.1
	HQC	200	195.8	7.2	97.9	-2.1
Doripenem	LQC	1	1.1	5.5	105.3	5.3
	MQC	87.5	90.0	3.1	102.9	2.9
	HQC	175	183.4	6.5	104.8	4.8
Levofloxacin	LQC	0.5	0.6	0.2	114.0	14.0

	MQC	15	15.9	1.6	106.1	6.1
	HQC	30	31.1	0.3	103.6	3.6
Ceftolozane	LQC	1	1.0	9.5	97.5	-2.5
	MQC	125	112.5	3.4	90.0	-10.0
	HQC	250	252.5	4.0	101.0	1.0
Tazobactam	LQC	1	1.1	2.0	107.0	7.0
	MQC	43.75	42.9	1.9	98.1	-1.9
	HQC	87.5	94.4	1.8	107.9	7.9
Tigecycline	LQC	1	1.1	2.5	105.1	5.1
	MQC	10	10.6	5.7	106.3	6.3
	HQC	20	22.2	1.8	111.0	11.0
Avibactam	LQC	0.5	0.5	1.3	103.4	3.4
	MQC	15	14.9	0.4	99.3	-0.7
	HQC	30	28.7	0.6	95.6	-4.4
Fosfomycin	LQC	10	10.6	3.4	106.3	6.3
	MQC	500	509.2	1.8	101.8	1.8
	HQC	1000	1014.3	0.7	101.4	1.4

Autosampler stability at 4 °C (15h)

Table S10. Stability of CAMHB extracted samples after 15h in autosampler

Compound	Level	Nominal Concentration (mg/L)	Fresh Concentration (mg/L)	(%) CV	Mean accuracy (%)	Deviation from nominal (%)
Ceftazidime	LQC	1	1.1	5.1	107.7	7.7
	MQC	75	83.2	1.6	111.0	11.0
	HQC	150	168.1	2.3	112.0	12.0
Cefepime	LQC	1	1.1	1.0	106.6	6.6
	MQC	100	114.1	1.9	114.1	14.1
	HQC	200	203.5	2.1	101.7	1.7
Piperacillin	LQC	1	1.1	1.7	112.7	12.7
	MQC	150	131.4	0.4	87.6	-12.4
	HQC	250	226.4	0.9	90.6	-9.4
Aztreonam	LQC	1	1.0	3.4	103.7	3.7
	MQC	150	157.1	0.3	104.7	4.7
	HQC	300	313.2	1.3	104.4	4.4
Meropenem	LQC	1	1.1	5.9	107.7	7.7
	MQC	100	100.8	4.4	100.8	0.8
	HQC	200	213.7	3.7	106.9	6.9
Doripenem	LQC	1	1.1	3.8	108.0	8.0
	MQC	87.5	97.4	3.1	111.3	11.3
	HQC	175	180.3	3.4	103.0	3.0
Levofloxacin	LQC	0.5	0.6	1.9	112.8	12.8

	MQC	15	15.6	1.4	103.9	3.9
	HQC	30	29.9	1.0	99.5	-0.5
Ceftolozane	LQC	1	1.1	8.0	107.2	7.2
	MQC	125	139.1	1.8	111.3	11.3
	HQC	250	280.0	0.9	112.0	12.0
Tazobactam	LQC	1	1.1	1.3	112.3	12.3
	MQC	43.75	47.1	1.2	107.7	7.7
	HQC	87.5	97.6	1.9	111.6	11.6
Tigecycline	LQC	1	1.0	2.4	101.8	1.8
	MQC	10	10.0	2.9	100.2	0.2
	HQC	20	20.6	6.6	103.2	3.2
Avibactam	LQC	0.5	0.5	1.0	108.3	8.3
	MQC	15	15.7	0.2	104.9	4.9
	HQC	30	32.3	0.4	107.5	7.5
Fosfomycin	LQC	10	10.7	2.2	107.4	7.4
	MQC	500	473.9	0.6	94.8	-5.2
	HQC	1000	956.0	0.6	95.6	-4.4

Long-term stability of various antibiotics in combination with Fosfomycin spiked in plasma

Cold storage stability at -30 °C (2 weeks)

Table S11. Stability after 2 weeks at -30 °C for drug combination plasma samples

Compound	Level	Nominal Concentration (mg/L)	Concentration after 2 weeks (-30 °C) (mg/L)	(%) CV	Mean accuracy (%)	Deviation from nominal (%)
Ceftazidime	LQC	1	1.0	9.4	97.8	-2.2
	MQC	75	78.7	1.2	105.0	5.0
	HQC	150	149.2	3.0	99.5	-0.5
Cefepime	LQC	1	1.0	2.7	98.6	-1.4
	MQC	100	87.2	1.7	87.2	-12.8
	HQC	200	178.5	4.7	89.3	-10.7
Piperacillin	LQC	1	1.1	1.0	112.6	12.6
	MQC	150	139.1	1.5	92.7	-7.3
	HQC	250	221.3	1.0	88.5	-11.5
Aztreonam	LQC	1	1.0	14.9	103.1	3.1
	MQC	150	135.4	1.7	90.2	-9.8
	HQC	300	291.8	1.5	97.3	-2.7
Meropenem	LQC	1	0.9	2.1	86.6	-13.4
	MQC	100	89.7	2.5	89.7	-10.3
	HQC	200	186.5	1.4	93.3	-6.7
Doripenem	LQC	1	0.9	3.7	91.0	-9.0
	MQC	87.5	84.9	6.7	97.0	-3.0
	HQC	175	171.5	2.9	98.0	-2.0
Levofloxacin	LQC	0.5	0.5	1.6	100.0	0.0
	MQC	15	14.8	1.3	98.7	-1.3
	HQC	30	31.3	1.4	104.3	4.3
Ceftolozane	LQC	1	0.9	6.5	86.9	-13.1
	MQC	125	108.0	2.0	86.4	-13.6
	HQC	250	216.3	0.2	86.5	-13.5

Tazobactam	LQC	1	1.1	1.1	107.9	7.9
	MQC	43.75	45.8	3.2	104.6	4.6
	HQC	87.5	92.8	0.6	106.0	6.0
Tigecycline	LQC	1	1.1	2.5	111.9	11.9
	MQC	10	9.9	2.4	98.9	-1.1
	HQC	20	20.5	2.2	102.5	2.5
Avibactam	LQC	0.5	0.5	1.6	100.6	0.6
	MQC	15	14.9	0.3	99.4	-0.6
	HQC	30	28.2	0.5	93.9	-6.1
Fosfomycin	LQC	10	10.2	6.5	102.2	2.2
	MQC	500	460.5	0.7	92.1	-7.9
	HQC	1000	984.3	1.7	98.4	-1.6

Cold storage stability at -30 °C (4 weeks)

Table S12. Stability after 4 weeks at -30° C for drug combination plasma samples

**Compound**	**Level**	**Nominal Concentration (mg/L)**	**Concentration after 1 month (-30 °C) (mg/L)**	**(%) CV**	**Mean accuracy (%)**	**Deviation from nominal (%)**
Ceftazidime	LQC	1	1.0	7.1	100.3	0.3
	MQC	75	78.8	2.6	105.0	5.0
	HQC	150	150.4	2.5	100.3	0.3
Cefepime	LQC	1	1.0	7.8	100.5	0.5
	MQC	100	97.4	1.9	97.4	-2.6
	HQC	200	179.3	3.2	89.6	-10.4
Piperacillin	LQC	1	1.1	1.5	106.0	6.0

	MQC	150	119.6	1.2	86.1	-13.9
	HQC	250	204.8	0.6	87.0	-13.0
Aztreonam	LQC	1	1.1	5.6	106.4	6.4
	MQC	150	144.4	1.7	96.2	-3.8
	HQC	300	288.4	3.1	96.1	-3.9
Meropenem	LQC	1	1.1	5.1	106.1	6.1
	MQC	100	93.8	5.2	93.8	-6.2
	HQC	200	183.6	1.8	91.8	-8.2
Doripenem	LQC	1	1.0	0.7	100.0	0.0
	MQC	87.5	90.2	1.3	103.1	3.1
	HQC	175	179.2	0.6	102.4	2.4
Levofloxa-cin	LQC	0.5	0.5	1.7	101.7	1.7
	MQC	15	15.1	1.2	100.9	0.9
	HQC	30	30.3	0.3	100.9	0.9
Ceftolozane	LQC	1	1.0	4.6	99.0	-1.0
	MQC	125	123.3	2.1	98.6	-1.4
	HQC	250	248.2	2.1	99.3	-0.7
Tazobactam	LQC	1	1.0	3.0	102.0	2.0
	MQC	43.75	43.2	0.6	98.7	-1.3
	HQC	87.5	84.4	1.3	96.4	-3.6
Tigecycline	LQC	1	1.0	5.1	97.8	-2.2
	MQC	10	10.3	2.4	102.8	2.8
	HQC	20	22.1	2.4	110.7	10.7
Avibactam	LQC	0.5	0.5	1.1	100.2	0.2
	MQC	15	13.9	0.3	92.6	-7.4
	HQC	30	26.2	0.9	87.3	-12.7
Fosfomycin	LQC	10	10.5	4.3	104.5	4.5
	MQC	500	462.0	0.8	92.4	-7.6
	HQC	1000	940.0	1.4	94.0	-6.0

Cold storage stability at -80 °C (2 weeks)

Table S13. Stability after 2 weeks at -80 °C for drug combination plasma samples

Compound	Level	Nominal Concentration (mg/L)	Concentration after 2 weeks (-80 °C) (mg/L)	(%) CV	Mean accuracy (%)	Deviation from nominal (%)
Ceftazidime	LQC	1	1.0	3.6	102.6	2.6
	MQC	75	75.8	1.7	101.0	1.0
	HQC	150	149.1	1.9	99.4	-0.6
Cefepime	LQC	1	1.0	6.1	102.0	2.0
	MQC	100	94.3	0.6	94.3	-5.7
	HQC	200	178.1	4.5	89.0	-11.0
Piperacillin	LQC	1	1.1	1.6	109.5	9.5
	MQC	150	139.6	1.0	93.0	-7.0
	HQC	250	221.6	2.0	88.6	-11.4
Aztreonam	LQC	1	0.9	2.5	87.6	-12.4
	MQC	150	141.8	2.1	94.5	-5.5
	HQC	300	291.7	3.7	97.2	-2.8
Meropenem	LQC	1	1.0	4.5	104.2	4.2
	MQC	100	95.0	1.4	95.0	-5.0
	HQC	200	210.7	3.2	105.4	5.4
Doripenem	LQC	1	1.0	4.7	100.6	0.6
	MQC	87.5	84.8	2.8	96.9	-3.1
	HQC	175	172.1	2.6	98.3	-1.7
Levofloxacin	LQC	0.5	0.6	0.9	109.4	9.4

	MQC	15	15.5	0.9	103.1	3.1
	HQC	30	32.7	0.4	109.1	9.1
Ceftolozane	LQC	1	0.9	6.3	91.1	-8.9
	MQC	125	109.7	3.3	87.7	-12.3
	HQC	250	215.8	0.3	86.3	-13.7
Tazobactam	LQC	1	1.1	2.6	108.8	8.8
	MQC	43.75	45.8	1.7	104.7	4.7
	HQC	87.5	83.5	0.5	95.4	-4.6
Tigecycline	LQC	1	1.1	3.8	109.8	9.8
	MQC	10	10.0	3.3	100.1	0.1
	HQC	20	21.6	3.8	107.8	7.8
Avibactam	LQC	0.5	0.5	1.9	105.1	5.1
	MQC	15	15.1	0.7	100.4	0.4
	HQC	30	28.6	0.5	95.2	-4.8
Fosfomycin	LQC	10	10.2	3.8	101.8	1.8
	MQC	500	472.9	0.2	94.6	-5.4
	HQC	1000	955.7	1.7	95.6	-4.4

Cold storage stability at -80 °C (4 weeks)

Table S14. Stability after 4 weeks at -80° C for drug combination plasma samples

Compound	Level	Nominal Concentration (mg/L)	Concentration after 1 month (-80 °C) (mg/L)	(%) CV	Mean accuracy (%)	Deviation from nominal (%)
Ceftazidime	LQC	1	1.0	9.8	98.8	-1.2
	MQC	75	77.3	2.1	103.0	3.0
	HQC	150	161.6	1.1	107.7	7.7
Cefepime	LQC	1	1.1	2.9	111.6	11.6
	MQC	100	101.3	9.1	101.3	1.3
	HQC	200	191.3	2.3	95.7	-4.3
Piperacillin	LQC	1	1.1	1.7	111.6	11.6
	MQC	150	125.9	0.9	88.9	-11.1
	HQC	250	208.2	1.3	86.6	-13.4
Aztreonam	LQC	1	1.0	4.2	104.2	4.2
	MQC	150	152.1	2.5	101.4	1.4
	HQC	300	293.3	2.2	97.8	-2.2
Meropenem	LQC	1	1.0	6.7	101.8	1.8
	MQC	100	95.2	3.0	95.2	-4.8
	HQC	200	192.2	2.9	96.1	-3.9
Doripenem	LQC	1	1.0	6.6	99.2	-0.8
	MQC	87.5	93.5	2.4	106.9	6.9
	HQC	175	186.1	1.7	106.3	6.3
Levofloxacin	LQC	0.5	0.5	3.2	100.6	0.6
	MQC	15	15.1	0.6	100.8	0.8
	HQC	30	30.6	0.3	102.0	2.0
Ceftolozane	LQC	1	0.9	2.1	89.3	-10.7
	MQC	125	122.5	3.0	98.0	-2.0

	HQC	250	251.9	3.4	100.8	0.8
Tazobactam	LQC	1	1.0	4.2	100.7	0.7
	MQC	43.75	44.3	2.7	101.3	1.3
	HQC	87.5	88.0	1.1	100.5	0.5
Tigecycline	LQC	1	1.0	2.5	96.9	-3.1
	MQC	10	10.5	1.6	105.0	5.0
	HQC	20	22.5	2.0	112.3	12.3
Avibactam	LQC	0.5	0.5	1.5	101.0	1.0
	MQC	15	14.2	0.5	94.5	-5.5
	HQC	30	27.9	0.3	92.9	-7.1
Fosfomycin	LQC	10	10.1	2.5	100.8	0.8
	MQC	500	453.0	1.6	90.6	-9.4
	HQC	1000	977.3	1.0	97.7	-2.3

Long-term stability of various antibiotics in combination with Fosfomycin spiked in CAMHB

Cold storage stability (2 weeks)

Table S15. Stability after 2 weeks at -30 °C for drug combination CAMHB samples

Compound	Level	Nominal Concentration (mg/L)	Concentration after 2 weeks (-30 °C) (mg/L)	(%) CV	Mean accuracy (%)	Deviation from nominal (%)
Ceftazidime	LQC	1	1.0	4.3	99.2	-0.8
	MQC	75	79.5	3.7	106.0	6.0
	HQC	150	146.5	2.8	97.6	-2.4
Cefepime	LQC	1	1.0	2.9	100.9	0.9
	MQC	100	102.0	3.9	102.0	2.0
	HQC	200	190.5	2.7	95.3	-4.7
Piperacillin	LQC	1	1.1	3.6	106.0	6.0
	MQC	150	134.3	3.5	89.5	-10.5
	HQC	250	222.4	1.5	89.0	-11.0
Aztreonam	LQC	1	1.0	6.0	102.2	2.2
	MQC	150	138.8	1.6	92.5	-7.5
	HQC	300	307.9	3.1	102.6	2.6
Meropenem	LQC	1	0.9	2.1	86.6	-13.4
	MQC	100	89.7	2.5	89.7	-10.3
	HQC	200	186.5	1.4	93.3	-6.7
Doripenem	LQC	1	0.7	6.8	73.1	-26.9[a]
	MQC	87.5	67.5	3.7	77.1	-22.9[a]
	HQC	175	144.2	0.9	82.4	-17.6[a]
Levofloxacin	LQC	0.5	0.5	1.3	108.1	8.1
	MQC	15	15.5	1.1	103.1	3.1
	HQC	30	30.5	1.1	101.7	1.7

Ceftolozane	LQC	1	0.9	3.4	91.3	-8.7
	MQC	125	118.4	3.4	94.7	-5.3
	HQC	250	233.1	2.8	93.2	-6.8
Tazobactam	LQC	1	0.9	4.6	93.8	-6.2
	MQC	43.75	39.6	3.1	90.4	-9.6
	HQC	87.5	84.8	2.2	96.9	-3.1
Tigecycline	LQC	1	1.0	2.1	98.8	-1.2
	MQC	10	8.7	1.5	87.3	-12.7
	HQC	20	17.4	1.0	87.2	-12.8
Avibactam	LQC	0.5	0.5	1.7	93.7	-6.3
	MQC	15	13.4	2.3	89.1	-10.9
	HQC	30	27.1	2.7	90.4	-9.6
Fosfomycin	LQC	10	10.3	6.1	103.1	3.1
	MQC	500	473.7	0.9	94.7	-5.3
	HQC	1000	985.3	1.5	98.5	-1.5

[a]Value does not meet acceptance criteria for stability according to FDA guideline

Cold storage stability (4 weeks)

Table S16. Stability after 4 weeks at -30° C for drug combination CAMHB samples

Compound	Level	Nominal Concentration (mg/L)	Concentration after 1 month (-30 °C) (mg/L)	(%) CV	Mean accuracy (%)	Deviation from nominal (%)
Ceftazidime	LQC	1	1.1	2.4	105.0	5.0
	MQC	75	75.3	2.5	100.4	0.4
	HQC	150	150.7	4.3	100.5	0.5
Cefepime	LQC	1	0.9	1.6	91.1	-8.9

	MQC	100	94.9	2.1	94.9	-5.1
	HQC	200	186.5	4.1	93.2	-6.8
Piperacillin	LQC	1	0.9	1.9	90.6	-9.4
	MQC	150	101.3	0.8	67.5	-32.5[a]
	HQC	250	174.0	1.7	69.6	-30.4[a]
Aztreonam	LQC	1	0.7	8.9	66.5	-33.5[a]
	MQC	150	137.8	3.2	91.8	-8.2
	HQC	300	277.5	3.2	92.5	-7.5
Meropenem	LQC	1	0.6	6.4	60.1	-39.9[a]
	MQC	100	63.5	2.4	63.5	-36.5[a]
	HQC	200	132.3	3.2	66.1	-33.9[a]
Doripenem	LQC	1	0.7	2.2	64.6	-35.4[a]
	MQC	87.5	53.0	3.4	60.6	-39.4[a]
	HQC	175	112.1	4.4	64.1	-35.9[a]
Levofloxa-cin	LQC	0.5	0.6	0.3	110.1	10.1
	MQC	15	15.9	0.5	106.3	6.3
	HQC	30	29.9	0.7	99.8	-0.2
Ceftolozane	LQC	1	0.9	7.2	86.1	-13.9
	MQC	125	113.0	0.3	90.4	-9.6
	HQC	250	226.9	2.3	90.8	-9.2
Tazobactam	LQC	1	0.7	8.4	69.7	-30.3[a]
	MQC	43.75	36.8	1.4	84.1	-15.9[a]
	HQC	87.5	79.6	1.5	91.0	-9.0
Tigecycline	LQC	1	0.9	2.2	87.0	-13.0
	MQC	10	10.1	9.0	100.6	0.6
	HQC	20	18.2	3.5	91.2	-8.8
Avibactam	LQC	0.5	0.4	0.6	82.3	-17.7[a]
	MQC	15	12.1	0.4	80.6	-19.4[a]
	HQC	30	24.2	0.1	80.6	-19.4[a]
Fosfomycin	LQC	10	10.4	1.3	103.6	3.6

MQC	500	477.0	1.6	95.4	-4.6
HQC	1000	946.3	0.7	94.6	-5.4

[a]Value does not meet acceptance criteria for stability according to FDA guideline

Cold storage stability (2 weeks)

Table S17. Stability after 2 weeks at -80 °C for drug combination CAMHB samples

Compound	Level	Nominal Concentration (mg/L)	Concentration after 2 weeks (-80 °C) (mg/L)	(%) CV	Mean accuracy (%)	Deviation from nominal (%)
Ceftazidime	LQC	1	1.0	2.2	104.3	4.3
	MQC	75	78.9	3.4	105.2	5.2
	HQC	150	154.5	4.2	103.0	3.0
Cefepime	LQC	1	1.1	2.4	108.8	8.8
	MQC	100	98.7	1.1	98.7	-1.3
	HQC	200	189.7	3.6	94.8	-5.2
Piperacillin	LQC	1	1.1	1.1	111.6	11.6
	MQC	150	139.0	1.0	92.6	-7.4
	HQC	250	223.9	1.1	89.6	-10.4
Aztreonam	LQC	1	1.0	8.7	101.3	1.3
	MQC	150	142.6	1.9	95.1	-4.9
	HQC	300	300.7	0.3	100.2	0.2
Meropenem	LQC	1	1.0	4.9	104.3	4.3
	MQC	100	97.3	2.5	97.3	-2.7
	HQC	200	199.3	5.5	99.6	-0.4

Doripenem	LQC	1	1.0	5.4	104.0	4.0
	MQC	87.5	90.9	4.5	103.9	3.9
	HQC	175	175.1	1.4	100.1	0.1
Levofloxa-cin	LQC	0.5	0.5	2.0	107.1	7.1
	MQC	15	15.3	1.5	102.1	2.1
	HQC	30	29.8	0.2	99.4	-0.6
Ceftolozane	LQC	1	0.9	5.5	91.4	-8.6
	MQC	125	117.8	0.8	94.3	-5.7
	HQC	250	235.3	0.2	94.1	-5.9
Tazobactam	LQC	1	1.0	8.3	103.8	3.8
	MQC	43.75	45.4	1.2	103.7	3.7
	HQC	87.5	95.3	1.8	108.9	8.9
Tigecycline	LQC	1	1.0	1.6	98.9	-1.1
	MQC	10	10.6	1.4	106.1	6.1
	HQC	20	20.5	2.5	102.3	2.3
Avibactam	LQC	0.5	0.5	0.6	101.2	1.2
	MQC	15	14.3	0.4	95.4	-4.6
	HQC	30	28.6	0.4	95.3	-4.7
Fosfomycin	LQC	10	11.1	2.0	110.6	10.6
	MQC	500	497.7	1.4	99.5	-0.5
	HQC	1000	963.0	1.6	96.3	-3.7

Cold storage stability (4 weeks)

Table S18. Stability after 4 weeks at -80° C for drug combination CAMHB samples

Compound	Level	Nominal Concentration (mg/L)	Concentration after 1 month (-80 °C) (mg/L)	(%) CV	Mean accuracy (%)	Deviation from nominal (%)
Ceftazidime	LQC	1	1.0	6.8	103.5	3.5
	MQC	75	79.8	4.3	106.4	6.4
	HQC	150	155.7	6.2	103.8	3.8
Cefepime	LQC	1	1.1	3.3	107.6	7.6
	MQC	100	102.8	2.2	102.8	2.8
	HQC	200	186.3	1.3	93.2	-6.8
Piperacillin	LQC	1	1.1	2.6	109.4	9.4
	MQC	150	135.9	2.0	90.6	-9.4
	HQC	250	219.0	2.8	87.6	-12.4
Aztreonam	LQC	1	1.1	2.6	109.7	9.7
	MQC	150	144.0	1.9	96.0	-4.0
	HQC	300	295.3	3.3	98.4	-1.6
Meropenem	LQC	1	1.0	4.7	97.9	-2.1
	MQC	100	95.4	3.7	95.4	-4.6
	HQC	200	194.7	6.3	97.4	-2.6
Doripenem	LQC	1	1.0	6.4	99.1	-0.9
	MQC	87.5	91.6	2.3	104.7	4.7
	HQC	175	185.2	2.4	105.8	5.8
Levofloxacin	LQC	0.5	0.6	1.4	111.1	11.1
	MQC	15	15.8	0.5	105.4	5.4

	HQC	30	31.5	0.4	104.9	4.9
Ceftolozane	LQC	1	0.9	4.4	87.5	-12.5
	MQC	125	120.3	3.8	96.2	-3.8
	HQC	250	234.8	2.9	93.9	-6.1
Tazobactam	LQC	1	1.1	6.0	105.4	5.4
	MQC	43.75	45.2	1.5	103.3	3.3
	HQC	87.5	93.1	0.2	106.4	6.4
Tigecycline	LQC	1	0.9	1.4	87.6	-12.4
	MQC	10	9.5	7.3	95.3	-4.7
	HQC	20	18.2	7.7	91.0	-9.0
Avibactam	LQC	0.5	0.5	0.5	103.0	3.0
	MQC	15	14.7	0.1	98.2	-1.8
	HQC	30	29.6	0.3	98.6	-1.4
Fosfomycin	LQC	10	10.5	0.8	104.7	4.7
	MQC	500	490.9	0.8	98.2	-1.8
	HQC	1000	1017.7	0.9	101.8	1.8
